# Untargeted urinary metabolomics for bladder cancer biomarker screening with ultrahigh-resolution mass spectrometry

**DOI:** 10.1038/s41598-023-36874-y

**Published:** 2023-06-16

**Authors:** Joanna Nizioł, Krzysztof Ossoliński, Aneta Płaza-Altamer, Artur Kołodziej, Anna Ossolińska, Tadeusz Ossoliński, Anna Nieczaj, Tomasz Ruman

**Affiliations:** 1 Faculty of Chemistry, Rzeszów University of Technology, 6 Powstańców Warszawy Ave., 35-959 Rzeszów, Poland; 2 Department of Urology, John Paul II Hospital, Grunwaldzka 4 St., 36-100 Kolbuszowa, Poland; 3 Doctoral School of Engineering and Technical Sciences at the Rzeszów University of Technology, 8 Powstańców Warszawy Ave., 35-959 Rzeszów, Poland

**Keywords:** Cancer, Urological cancer, Bladder cancer, Mass spectrometry, Biomarkers

## Abstract

Bladder cancer (BC) is a common urological malignancy with a high probability of death and recurrence. Cystoscopy is used as a routine examination for diagnosis and following patient monitoring for recurrence. Repeated costly and intrusive treatments may discourage patients from having frequent follow-up screenings. Hence, exploring novel non-invasive ways to help identify recurrent and/or primary BC is critical. In this work, 200 human urine samples were profiled using ultra-high-performance liquid chromatography and ultra-high-resolution mass spectrometry (UHPLC-UHRMS) to uncover molecular markers differentiating BC from non-cancer controls (NCs). Univariate and multivariate statistical analyses with external validation identified metabolites that distinguish BC patients from NCs disease. More detailed divisions for the stage, grade, age, and gender are also discussed. Findings indicate that monitoring urine metabolites may provide a non-invasive and more straightforward diagnostic method for identifying BC and treating recurrent diseases.

## Introduction

Cancer is one of the humanity’s most significant problems in the twenty-first century that also occupy thousands of scientists. Cancer is the leading cause of death among people under 70. Recent trends show that cancer may be the leading cause of premature death in most countries this century^1^. Urological cancers constitute a large part of all types of cancers worldwide. Their incidence and mortality are still increasing, which places a significant burden on healthcare worldwide^1^. The early detection of cancer contributes to early diagnosis and subsequent treatment^2^.

Bladder cancer (BC) is one of the most common urinary tract cancers affecting men and women^3^. The incidence of this cancer depends mainly on age, sex, carcinogenic factors, diet, and alcohol consumption or smoking^4^. Based on the histological classification, several types of bladder cancer were classified, including non-muscle invasive bladder cancer (NMIBC), which accounts for about 70–85% of all bladder tumors, and muscle-invasive BC (MIBC). NMIBC comprises noninvasive papillary carcinomas (pathologic stage Ta), submucosal invasive tumors (T1), and carcinoma in situ (CIS). MIBC contains tumors that have spread into muscle (stage T2), perivisceral fat (stage T3), or adjacent organs (stage T4). Histology classifies BC as low-grade (LG) tumors that seldom expand from their source location and high-grade (HG) tumors that are more aggressive and invasive. Moreover, about 50% of NMIBC cases aft, after all, recur despite radical treatment, and about 30% experience disease progression to MIBC^5^. This is why cancer patients are screened mainly for recurrence of the disease and metastasis of the disease to other sites^3^.

Transurethral resection of bladder tumor (TURBT), occasionally followed by intravesical instillation of mitomycin or Bacillus Calmette-Guerin (BCG) therapy, is the standard first-line treatment for early BC. The conventional therapy for MIBC, on the other hand, is a radical cystectomy with pelvic lymph node dissection. This is used with neoadjuvant or adjuvant cisplatin-based chemotherapy^6^. Despite such rigorous therapy, BC patients have a dismal survival rate. It is widely known that the sooner the cancer is detected, the greater the chance of treating the patient^7^. Some of the varieties of cancer are undetectable at an early stage using cystoscopy. Incredibly, flat, non-invasive with high-grade cancer is practically invisible in cystoscopy. Moreover, it is often mistakenly interpreted as a common inflammatory area because of its appearance. Therefore, metabolomics can be the most suitable way to achieve this. Due to the direct contact of the tumor with the urine, specific disease biomarkers may be present in this fluid. In recent years, metabolomics research in diagnosing and understanding numerous illnesses has increased dramatically^8^.

Several analytical approaches have been developed to understand better the metabolic alterations in biological systems, including cancer phenotypic changes. Nevertheless, two analytical platforms—nuclear magnetic resonance (NMR)^9^ spectroscopy and mass spectrometry (MS), which are frequently combined with liquid chromatography (LC)^10^, provide the complete screening of cancer metabolomes. MS, compared to NMR, detects far wider variety of molecules with much greater sensitivity, resolution, and accuracy using much less material^11^. During the last fifteen years, metabolomic analytical approaches have been extensively employed to explore BC and find possible biomarkers in urine, serum, and tissues^4,5,12,13^. Compared to serum and tissue, urine metabolomics may be affected by dilution factor, but urine is more available than tissue or serum, and the procedure non-invasive. Recently, many detailed articles have been published on potential urinary markers of BC, demonstrating significant interest in this field^14,15^. Unfortunately, none of the biomarkers invented and tested so far guarantee 100% detection of cancer at an early stage. Even though their detection characteristics are still high, they are associated with enormous costs that the global health service cannot afford^16^. This is still desirable for scientists to research subsequent biomarkers, thanks to which we will increase the percentage of cases with early detection of bladder cancer.

Most investigations of urine from patients with bladder cancer were based on NMR^17^ or mass spectrometry coupled to liquid chromatography (LC)^18–21^ and gas chromatography (GC)^22,23^. One of the first report of metabolomic profiling of urine from BC patients using high-performance liquid chromatography coupled with mass spectrometry appeared in 2008^24^. The study was conducted on samples from 41 bladder cancer patients and 48 healthy individuals. The results indicated that metabolomics using HPLC–MS had the potential to become a noninvasive early detection test for BC. Similar conclusions were drawn in 2011 by Huang and coworkers, who indicated fourteen compounds differentiating these two groups based on the urine analysis of 27 patients with BC and 32 healthy volunteers^25^. In the same year, urine profiling with external validation was performed by Putluri and coworkers^26^ on a much larger group of 85 BC patients and 51 controls, indicating 35 potential BC biomarkers. In 2013, two more urine profiling works were published that were based on a relatively small groups of BC patients^27,28^. The first profiling of urine metabolites of a larger group of 138 BC patients and 121 controls using HPLC-QToF-MS was performed in 2014 by Jin and coworkers^29^. The research identified 12 differential metabolites that may be useful for the distinction between the BC and control groups. However, mentioned research was based on mass spectrometer of 20,000 resolution that is three-times lower value that for instrument used in our report. Also, authors used 2.6 micron HPLC column that have inferior resolving power compared to our 1.7 micron one. In later years, several publications indicated potential small-molecule biomarkers for early detection of bladder cancer; however^30–33^, to our knowledge, only two papers using a group of more than one-hundred patients and with external validation have been published so far^16,20^. Similarly, there are very few reports on the analysis of urine of patients with BC, considering the division into different stages and grades of cancer, as well as gender and age^21,34^. There are no reports published that combine large cohorts of patients and also controls with ultrahigh performance liquid chromatography combined with ultrahigh resolution mass spectrometry system.

In this work, we report the results of an untargeted analysis of human urine with ultra-high-resolution mass spectrometry coupled with ultra-high-performance liquid chromatography (UHPLC-UHRMS) with external validation. This study employed a large number of patients—100 cancer patients and 100 controls. The untargeted analysis focused on urine metabolic changes generated by bladder cancer and stratified the disease by stage, grade, age, and gender. Our study reveals potential urinary BC biomarkers for early detection, screening, and differential diagnosis.

## Materials and methods

All chemicals were of LCMS- or analytical reagent-grade. Deionized water (18 MΩ cm) was produced locally. LC–MS-grade methanol was bought from Sigma Aldrich (St. Louis, MO, USA).

### Instrumentation

The untargeted analysis was performed using a Bruker Elute UHPLC system with Hystar 3.3 software and an ultra-high-resolution mass spectrometer Bruker Impact II (60,000 + resolution version; Bruker Daltonik GmbH) ESI QTOF-MS with Data Analysis 4.2 (Bruker Daltonik GmbH) and Metaboscape (ver. 2022b). Metabolite separation was achieved with a gradient of mobile phases using a Waters UPLC column ACQUITY BEH (C18 silica, 1.7 μm particles, 50 × 2.1 mm) with a compatible column guard was used for all analyses. Further details are described in our previous publication^35^ and supplementary information 1 (section S1).

### Collection of human urine samples

Urine samples were taken from 100 BC patients at John Paul II Hospital in Kolbuszowa, Poland (average age 73, white ethnicity). Control group consisted of age and sex matched patients admitted to the Urology Department for surgery of benign urological conditions including benign prostatic hyperplasia (BPH), urine stones, phimosis, UPJO (Ureteropelvic Junction Obstruction), and stress urinary incontinence. Prior to the procedure (day before) each patient, underwent a comprehensive set of laboratory tests, including a blood count, electrolyte analysis, coagulation panel, creatinine measurement, glomerular filtration rate (GFR) assessment, urinalysis, lung X-ray, and ultrasound of the cavity as a part of standard protocol before each surgery. After extensive clinical questioning and laboratory testing, all patients with cancer had transurethral resection of bladder tumor (TURBT). The study was authorized by the University of Rzeszow's local Bioethics Committee (Poland, permit number. 2018/04/10) and complied with relevant rules and legislation. All methods were performed in accordance with the relevant guidelines and regulations. Written informed consent was obtained from all subjects. All patients who participated in the trial were told about the study’s goal and methods, and they completed an informed permission form. Patients hospitalized in the urology department for surgical treatment of benign urological diseases include the whole NCs group (urolithiasis, benign prostate hyperplasia, testicular hydrocele, varicocele, phimosis, ureteropelvic junction stenosis, urinary incontinence, urethral stricture). Each individual has received at least one abdominal ultrasound to rule out neoplasms (patients with urolithiasis frequently get a CT scan) and a basic set of lab tests necessary for urological surgery to rule out inflammation. Patients in the control group received written authorization to give leftover urine for investigation after being informed about the research program. Each participant had 10 ml of urine collected from them. At room temperature, samples were centrifuged at 3000 rpm for 10 min. The samples were then stored at − 80 °C until use. The clinical characteristics of the patients are presented in supplementary information 1, table S1.

### Sample preparation

As detailed in our recent work, medium-to-high polarity metabolites were isolated from urine samples^36^. In summary, urine samples were thawed at 4 °C and then centrifuged at 12,000 × *g* for 5 min at 4 °C. A total of 900 µL of acetone was added to 300 µL of supernatants. After vortexing for 1 min, the solutions were incubated at room temperature for 20 min, followed by 20 min at − 20 °C, and then centrifuged at 6000 × *g* for 5 min at 4 °C. Then, 800 µL of supernatants were transferred to a fresh polypropylene tube. The pellets were resuspended in 500 µL of an acetone-H_2_O (3:1 v/v) combination and vortexed extensively. Samples were centrifuged at 12,000 × *g* for 10 min at 4 °C. The supernatants from the pellet washes were mixed with those from the first spin. 260 µL of mixed supernatants were vacuum dried in a speedvac-type concentrator, dissolved in 900 µL of methanol, vortexed, and centrifuged (12,000 × *g* for 5 min at 4 °C). A 800 µL supernatant volume was placed into an HPLC vial and put into the Elute autosampler.

### Data analysis

In this study, we characterized the metabolic profile of urine from 100 patients with diagnosed BC and also from 100 normal control subjects (NCs) to develop potentially discriminant biomarkers for early, specific, and sensitive detection of this disease using ultra-high-resolution LC–MS. Two datasets from BC patients and NCs have been created: a training set, which contained 70% of all samples, and a validation set, which had 30% of all samples. In the training set, samples from a patients with certain stages and grades of BC made up 80% of all samples for a particular stage and grade of this disease. On the two datasets, urine metabolic profiling was carried out separately. The training data set was used to identify urine diagnostic markers differentiating the control group from cancer, high- and low-grade, pTa and pT1 stage. In turn, the validation set was used to validate the diagnostic performance of urine metabolite biomarkers independently. In the case of the analysis of samples from patients with pT2 stage of BC, in different age groups, and from women, the number of samples was insufficient to conduct a reliable statistical analysis divided into two independent sets. Therefore, the study was performed for the entire data set. In comparing patients of different sex and age, the control group consisted of people of a given sex and from a specific age group.

### Multivariate statistical analysis

For raw data, we have used Metaboscape v.2022b program recommended filtration of recorded features that removes the ones that for given *m/z* and retention time are not detected in samples from minimum 10 patients. This amount of patients is correlated with smallest group of given medical condition. Then data were exported and saved in CSV format. Subsequently, the data was imported into the Metaboanalyst 5.0 online software^37^ for further analysis. Within the Metaboanalyst platform, the data was normalized using log-transformed, auto-scaled, and sum-normalized before analysis. The resulting metabolite profiles were then submitted to unsupervised Principal Component Analysis (PCA). The separation identified in the 2D and 3D PCA score plots between the BC and control groups was further investigated utilizing supervised multivariate statistical analysis such as Orthogonal Partial Least Squares Discriminant Analysis (OPLS-DA). The goodness of fit (R^2^Y) and predictive ability of the OPLS-DA models were used to evaluate their quality (Q^2^). VIP plots were created to identify the metabolites most substantially responsible for group separation. VIP values of more than 1.0 were considered promising biomarker candidates. Permutation tests with 2000-fold repetition were used to assess the correctness of the multivariate statistical models and rule out the possibility that the observed separation in the OPLS-DA is attributable to chance (*P*-value < 0.05). The t-test with Mann–Whitney and Bonferroni correction was used to determine the statistical significance of metabolite level differences. Less than 0.05 *P*-values and false discovery rates (FDR; q-value) were considered statistically significant. The diagnostic value of the identified metabolites was evaluated using receiver operating characteristic curve (ROC) studies and random forest modeling. The metabolites' performance was calculated using the area under the curve (AUC), 95% confidence interval, specificity, and selectivity. AUC values greater than 0.9 indicate that the model is highly dependable, AUC values between 0.7 and 0.9 suggest moderate reliability, AUC values between 0.5 and 0.7 indicate low reliability, and AUC 0.5 shows that the model prediction is no better than chance. Only variables with an AUC greater than 0.70 were deemed meaningful. The training and validation datasets were subjected to separate multivariate statistical analyses. Chemicals that distinguish between tumor and control urine samples were chosen via external validation, which involves using two different datasets (here referred to as the training and validation datasets) to validate the performance of a model. The final collection of possible BC biomarkers met all testing and validation data set requirements. Chemometric methods such as 2D PCA, OPLS-DA, and ROC analysis were also employed to compare and contrast metabolic profiles between various grades and stages of bladder cancer. A metabolic pathway impact study was performed in MetaboAnalyst 5.0 utilizing the Kyoto Encyclopedia of Genes and Genomes (KEGG) pathway library for Homo sapiens^38^ to discover metabolic pathways influenced by bladder cancer. The Small Molecule Pathway Database performed quantitative pathway enrichment analysis (SMPD). Each affected pathway was identified using statistical *P*-values, Holm p (*P*-value corrected using the Holm-Bonferroni technique), and FDR (*P*-value adjusted using the False Discovery Rate), computed using pathway topology analysis.

### Ethical approval

The local Bioethics Committee approved the study protocol at the University of Rzeszow (Poland) (permission no. 2018/04/10). Written informed consent was obtained from all subjects and/or their legal guardian(s).

## Results

### Distinguishing between bladder cancer and control urine samples

**Figure 1:**
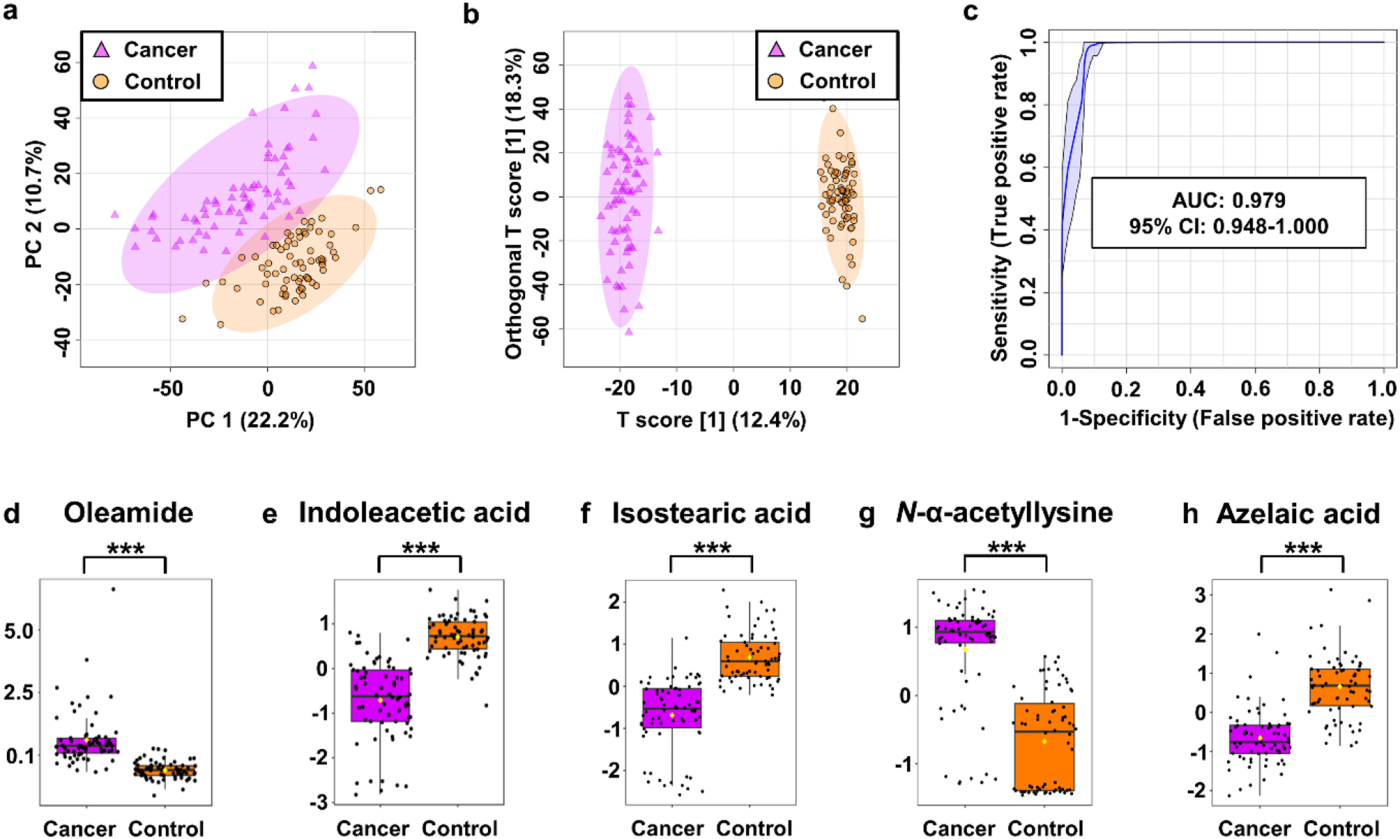
Metabolomic analysis of BC and NC urine samples in the training set. (**a**) PCA and (**b**) OPLS-DA score plots of tumor (violet) and control (orange) urine samples. (**c**) The receiver operator characteristic (ROC) curves. (**D**–**G**) The box-and-whisker plots of selected metabolites were observed in the control and BC urine samples.

In total, 2969 m*/z* features were detected in each urine sample, with the condition that feature is found in at least nine samples corresponding to the smallest group of cancer subtype. Both subsets' unsupervised 2D PCA score plots clearly distinguished between cancer patients and controls. The principal components 1 and 2 (i.e., PC1 and PC2), which accounted for 22.2% and 10.7%, respectively, provided the best group separation in the training set. In the middle 95% of the field of view, just a few outliers were found (Fig. 1a). Additionally, in the validation set, PC1 (26.2%) and PC2 (9.4%) showed the best separation between cancer and control urine samples (Fig. S1A, information 1).

To investigate the metabolic differences between the BC and NC groups, a supervised multivariate OPLS-DA analysis was performed. The score plot in the training set showed a clear divergence between the two groups (Fig. 1b). The OPLS-DA model was validated using 2000 permutation tests (Table S2, supplementary information). There was good discrimination between the two groups (Q^2^ = 0.960, R^2^Y = 0.991, *p*-value 5E-04 (0/2000)), revealing significant differences in the metabolic profiles of cancer urine samples versus control urine samples. This OPLS-DA model has a high R^2^Y and Q^2^, indicating good interpretability and predictability. A similar tendency to discriminate BC patients and NCs was observed in the validation set's OPLS-DA model (Table S2), which was confirmed by the excellent permutation test results (Q^2^ = 0.918, R^2^Y = 0.984, *p*-value 5E-04 (0/2000)). Volcano plot and PCA biplot of the most significant metabolite changes comparing cancer and control group was shown in supplementary information in the Figure S2. The VIP plot generated by the OPLS-DA model was used to select potential urine bladder cancer biomarkers. Then, univariate ROC analysis was performed on both the training and validation sets to assess the models' diagnostic ability. The area under the ROC curve (AUC), an adequate measure of model performance, was utilized as a metric to analyze the biomarkers' sensitivity and specificity. Only *m/z* with an AUC value higher than 0.70 were considered to be relevant by combining the VIP (> 1.0) and AUC (> 0.7) with the independent t-test results (p-value and FDR from t-test under 0.05), 464 variables in the training set were chosen as differential in urine for BC patients and NCs. In turn, 548 variables were considered significant in the validation set. Finally, 51 m*/z* features common to both sets were left for which a specific chemical compound was assigned (Table 1, supplementary information 2). The results showed that in urine samples, 5 of the previously selected 51 metabolites have a very high AUC value of more than 0.9 and high parameters of specificity and sensitivity of more than 80 and 81%, respectively (Table 1 and S1, supplementary information 2). The combination of mass features in the validation and training set was a robust discriminator of control versus bladder cancer urine samples (AUC > 979%), as illustrated in Fig. 1c and S1C.

**Table 1:** Differential metabolites for discrimination between BC patients and NCs (*P*-value and FDR < 0.001; VIP > 1; FC < 0.5 and > 2; AUC > 0.9).

No.	Name		Formula	m/z^a^	RT [min]	VIP^b^	FC^c^	*P*-value	FDR	AUC	Spec. [%]^d^	Sens. [%]^d^
1	Oleamide^e,g,h^	Cancer versus control	C_18_H_35_NO	282.2790	5.08	1.70	3.00	2.34E−20	1.73E−18	0.953	90	89
2	Indoleacetic acid^e,f,g^		C_10_H_9_NO_2_	217.0974	2.02	2.08	0.15	1.14E−19	5.43E−18	0.944	90	90
3	Isostearic acid^e,g,h^		C_18_H_36_O_2_	285.2785	0.20	1.95	0.16	1.28E−19	5.99E-18	0.944	86	89
4	*N*-Alpha-acetyllysine^e,f,g^		C_8_H_16_N_2_O_3_	268.1056	0.14	1.96	14.89	4.17E−17	9.94E-16	0.912	80	90
5	Azelaic acid^e,f,g,h^		C_9_H_16_O_4_	171.1014	2.54	1.94	0.35	2.97E−16	6.01E−15	0.900	87	81
6	Indoleacetic acid^e,f,g^	LG versus control	C_10_H_9_NO_2_	217.0974	2.02	2.12	0.18	1.16E−13	4.19E−12	0.934	87	96
7	Isostearic acid^e,g,h^		C_18_H_36_O_2_	285.2785	0.20	1.97	0.18	4.91E−13	1.50E−11	0.923	89	82
8	Isostearic acid^e,g,h^	HG versus control	C_18_H_36_O_2_	285.2785	0.20	1.94	0.13	1.64E−13	1.43E−11	0.967	97	87
9	Oleamide^e,g,h^		C_18_H_35_NO	282.2790	5.08	1.73	4.65	2.88E−13	1.85E−11	0.962	96	87
10	Indoleacetic acid^e,f,g^		C_10_H_9_NO_2_	217.0974	2.02	2.12	0.12	5.02E−13	2.75E−11	0.958	96	93
11	2-Furoylglycine^e,f,g,h^		C_7_H_7_NO_4_	170.0447	1.60	2.00	0.11	7.81E−13	3.66E−11	0.954	96	87
12	Azelaic acid^e,f,g,h^		C_9_H_16_O_4_	171.1014	2.54	1.87	0.27	1.87E−12	7.16E−11	0.946	90	87
13	*Cis*,*cis*-Muconic acid^e,f,h^		C_6_H_6_O_4_	125.0232	1.53	1.97	0.09	3.03E−12	1.08E−10	0.942	91	87
14	Phenylglyoxylic acid^e,f,g^		C_8_H_6_O_3_	151.0386	1.85	1.89	0.18	5.16E−12	1.62E−10	0.937	96	83
15	*N*-Alpha-acetyllysine^e,f,g^		C_8_H_16_N_2_O_3_	268.1056	0.14	1.85	15.22	1.47E−11	3.85E−10	0.928	100	80
16	Methylmalonic acid^e^		C_4_H_6_O_4_	119.0344	0.03	2.12	0.13	1.55E−11	3.95E−10	0.927	93	90
17	3,4-Dihydroxymandelic acid^e^		C_8_H_8_O_5_	226.0709	1.85	1.79	0.19	4.10E−11	8.36E−10	0.918	90	83
18	*N*-Acetylserotonin^e,f^		C_12_H_14_N_2_O_2_	175.1227	1.75	1.81	0.21	8.73E−11	1.57E−09	0.911	87	90
19	3-Hydroxy-4-methoxycinnamic aicd^e^		C_10_H_10_O_4_	195.0654	1.79	1.87	0.15	1.06E−10	1.87E−09	0.909	86	87
20	Tiglylglycine^e,f^		C_7_H_11_NO_3_	199.1076	1.56	1.69	0.32	1.66E−10	2.74E−09	0.905	81	87

^a^Experimental monoisotopic mass of ion; ^b^VIP scores derived from OPLS-DA model; ^c^fold change between cancer and control serum calculated from the abundance mean values for each group – cancer-to-normal ratio; ^d^ROC curve analysis for individual biomarkers; ^e^the metabolites identified by high precursor mass accuracy; ^f^the metabolites identified by matching retention time; ^g^the metabolites identified by matching isotopic pattern; ^h^the metabolites identified by matching MS/MS fragment spectra; AUC: area under the curve; FC: fold change; FDR: false discovery rate; *m/z*: mass-to-charge ratio; RT: retention time; Sens.: Sensitivity; Spec.: Specificity; VIP: variable influence on projection.

### Determination of low- and high-grade bladder cancer and control urine samples

Another series of PCA and OPLS DA analyses were performed on the training (70 NCs, 30 patients with HG, and 38 patients with LG) and validation (30 NCs, 12 patients with HG, and 17 patients with LG) data sets (Tabe S1) to see if metabolomics analysis of urine samples could help discriminate between different grades of BC. Patients with PLUMP were excluded from this analysis due to their small number.

**Figure 2:**
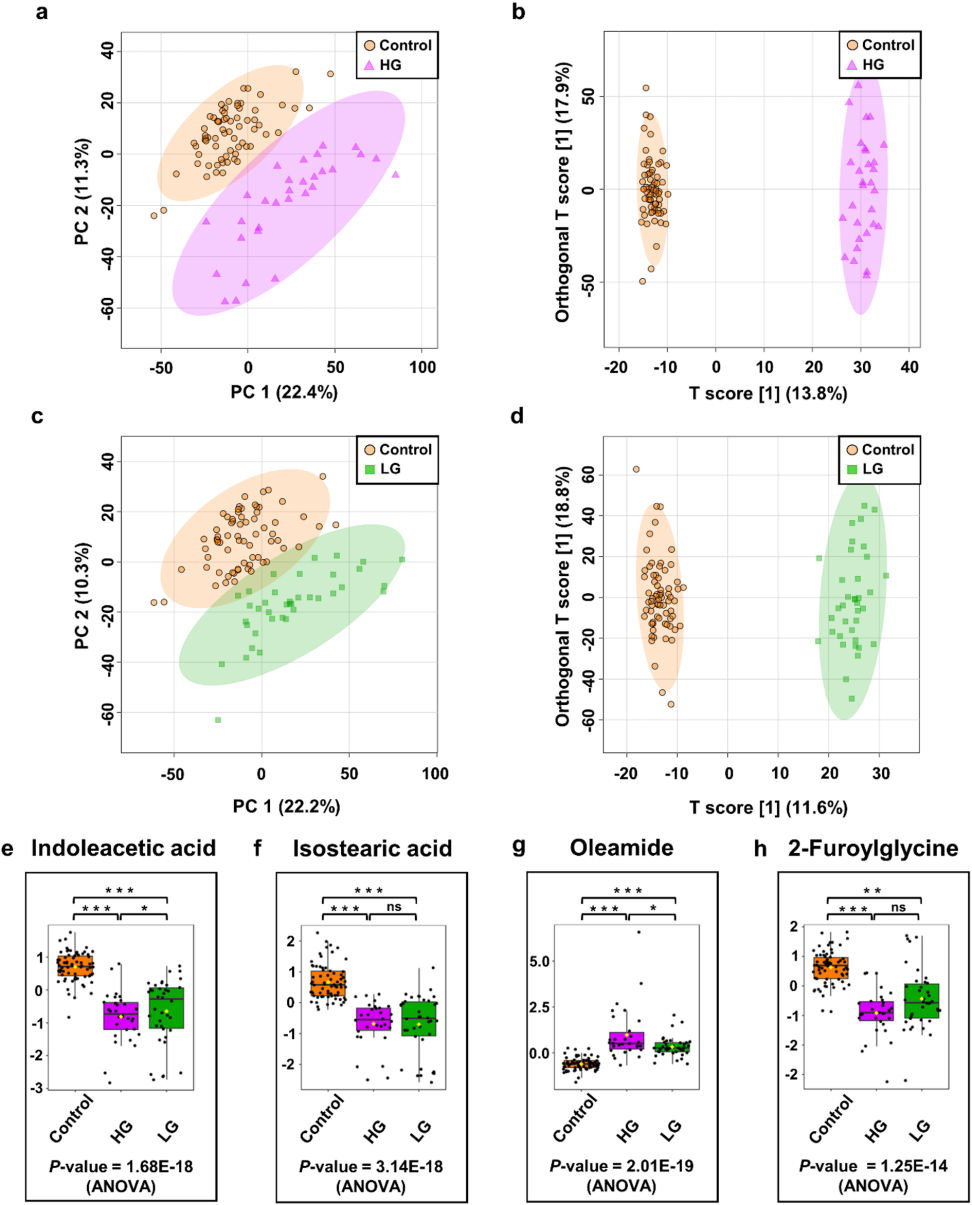
Metabolomic analysis of HG/LG BC and NCs of urine samples in the training set. (**a**) PCA and (**b**) OPLS-DA score plots of HG BC (violet) and control (orange) urine samples. (**c**) PCA and (**d**) OPLS-DA score plots of LG BC (green) and control (orange) urine samples. (**e**, **h**) The box-and-whisker plots of selected metabolites were observed in the control, HG, and LG BC urine samples.

In both the training and validation sets, PCA and OPLS-DA scores plots showed good separation between control groups and cancer groups with different grades of tumors (LG vs. NCs and HG vs. NCs) (Fig. 2a–d, S3). The quality factors for these models were Q^2^ > 0.879 and R^2^Y > 0.983, and the *P*-values from permutation tests (n = 2000) were less than 5E−4 (Table S3), which means that the metabolites profiles of these two groups could not be more different. But in the PCA scores plot, we didn't see a big difference between the LG and HG BC patients (data not shown). In the LG BC vs. NCs OPLS-DA model, 26 identified chemical compounds were considered significant (VIP > 1, *P*-value 0.05) in both the training set and the validation set (Table S2, supplementary information 2).

Analysis of HG BC vs. NCs in the training and validation sets of the OPLS-DA model showed that 63 commonly identified compounds were important in separating the two groups (Table S3, supplementary information 2). Based on the results of univariate ROC curve analyses, it was determined that these models have satisfactory diagnostic performance. Two of the twenty-six metabolites in the LG versus NCs model and thirteen of the sixty-three in the HG vs. NCs model had AUC values higher than 0.90 with sensitivity and specificity of more than 80 and 87%, respectively (Table 1). Selected metabolites most differentiating different grades of BC and NCs are shown in Fig. 2e–h.

### Determination of different stages of bladder cancer and control urine samples

**Figure 3:**
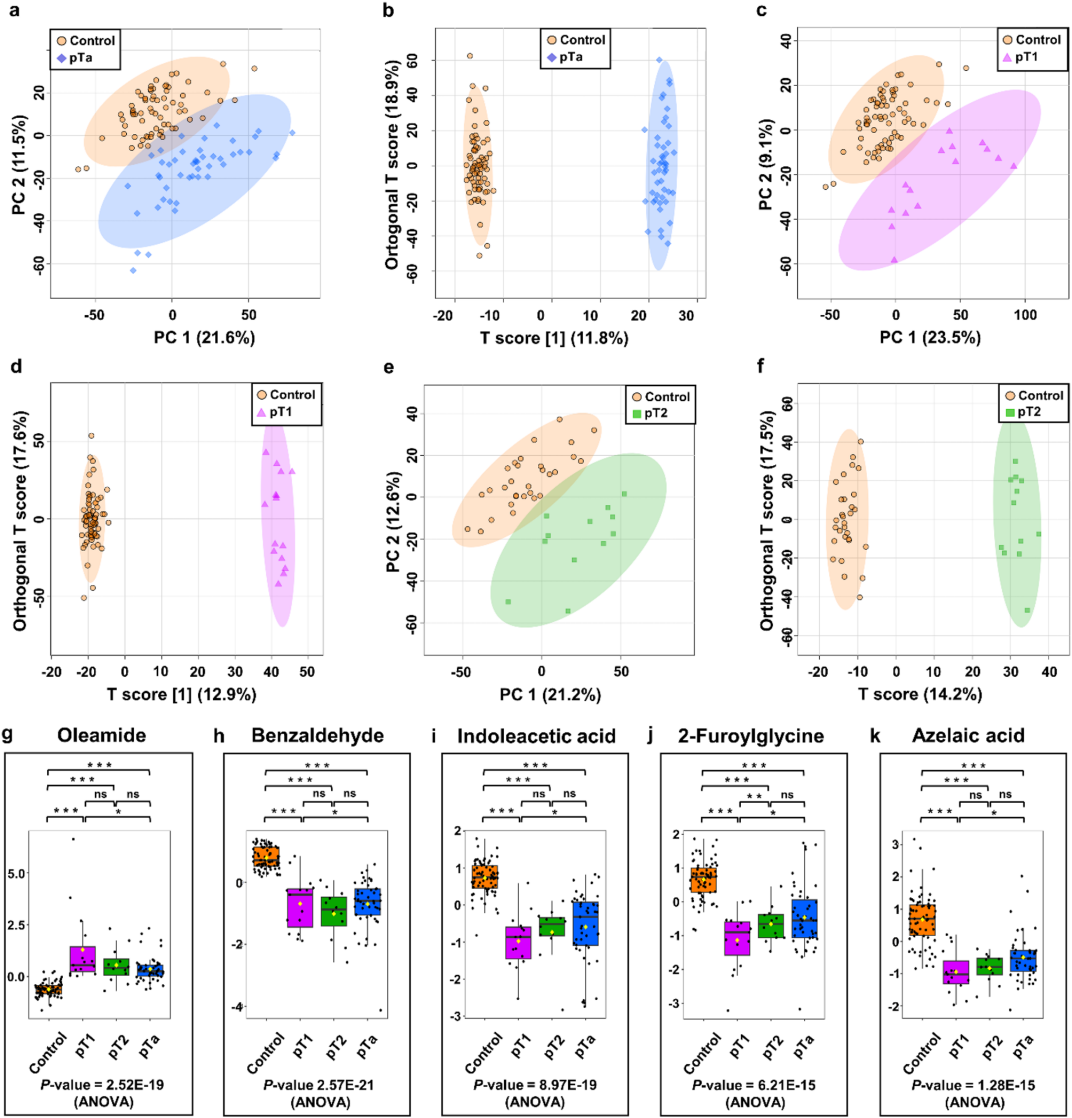
Metabolomic analysis of pTa/pT1/pT2 BC and NCs of urine samples in the training set. (**a**) PCA and (**b**) OPLS-DA score plots of pTa BC (blue) and control (orange) urine samples. (**c**) PCA and (**d**) OPLS-DA score plots of pT1 BC (violet) and control (orange) urine samples. (**e**) PCA and (**f**) OPLS-DA score plots of pT2 BC (green) and control (orange) urine samples. (**g**–**k**) The box-and-whisker plots of selected metabolites were observed in control, pTa, pT1, and pT2 BC urine samples.

To differentiate between the various stages of bladder cancer, PCA and OPLS-DA models were developed. The 68 urine samples from patients with noninvasive papillary carcinomas (pTa) were divided into training (70 NCs, 47 patients with pTa) and validation (30 NCs, 21 patients with pTa) sets. The 19 urine samples from patients with the pT1 stage of BC were divided into training (70 NCs, 15 patients with pT1) and validation (30 NCs, 5 patients with pT1) sets. In the case of the pT2 stage of BC, the analysis was performed without dividing it into two sets (30 NCs, 12 patients with pT2). The PCA and OPLS-DA score plots demonstrated a decent separation between NCs and the various stages of BC (pTa vs. NCs, pT1 vs. NCs, and pT2 vs. NCs, Fig. 3 a-f).

Quality factors for these models were Q^2^ > 0.836 and R^2^Y > 0.985, and *P*-values derived from permutation tests (n = 2000) were less than 5E-4 (Table S2), suggesting very strong discrimination of metabolite profiles between these groups. The performance of three models in differentiating between pTa, pT1, and pT2 bladder cancer stages and NCs was then evaluated using ROC curve analysis. Based on the cut-off criteria (FC > 2 < 0.5, VIP > 1; AUC > 0.7, *P*-value and FDR < 0.05), finally, 19, 68, and 81 chemical compounds appeared to be most relevant for sample distinction between pTa BC vs. NCs, pT1 BC vs. NCs, and pT2 BC vs. NCs, respectively (Table S4-S6, supplementary information 2). Comparing the three cancer stage groups (pT1 versus pTa versus pT2) revealed no statistically significant differences (data not shown). Selected metabolites most differentiating different stages of BC and NCs are shown in Fig. 3g–k.

### Sex-related differentiation of metabolomic profiles

The differentiation of metabolites in urine extracts from patients of different sexes was studied. The control group consisted of people matched to a given sex. The comparison of the male BC patients with the male control group was performed with a division into the training (55 male BC, 51 male control) and validation (26 male BC, 19 male control) sets. The group of female patients was compared on the whole data set (19 female BC, 30 female control).

**Figure 4:**
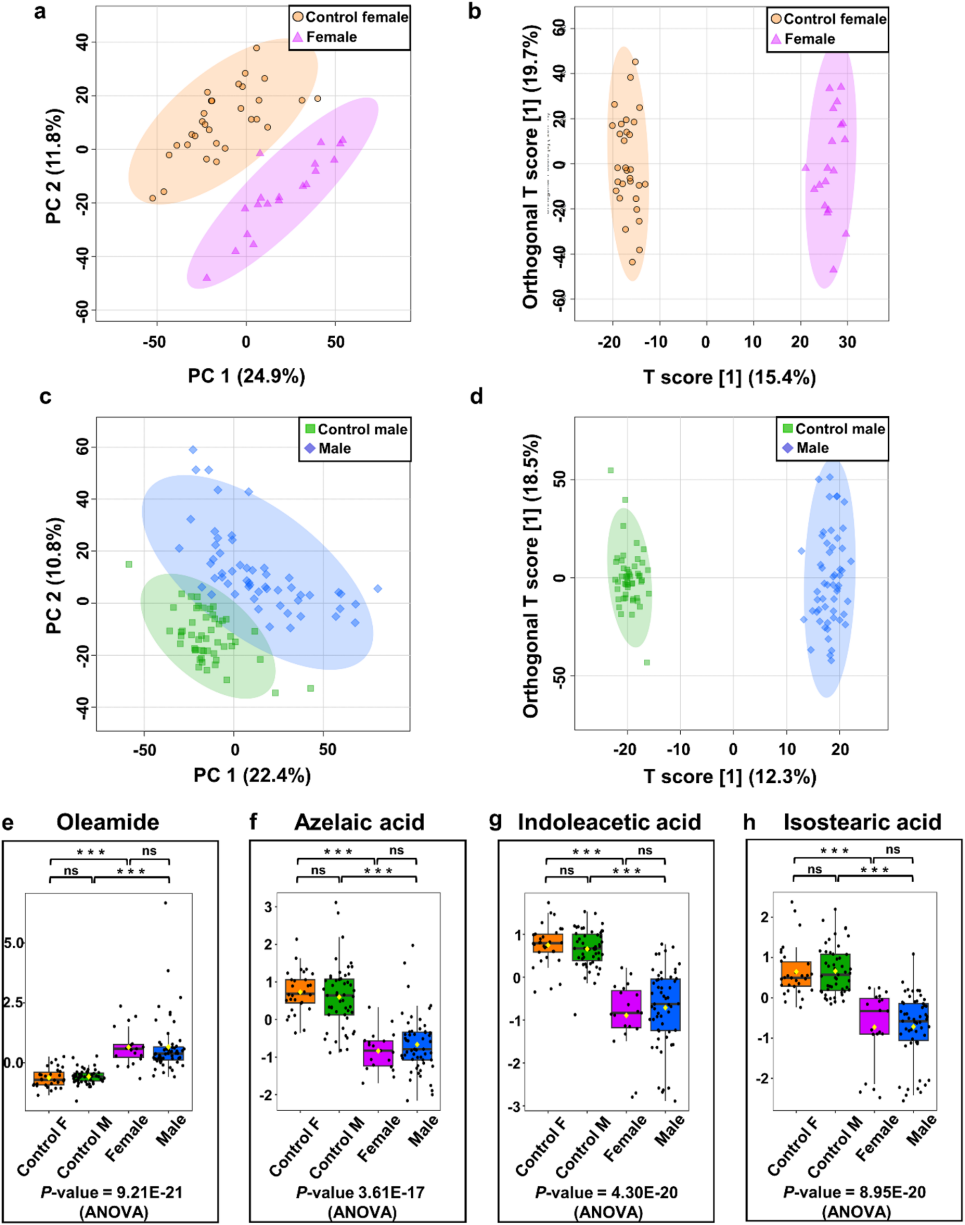
Metabolomic analysis of female/male BC and NCs of urine samples. (**a**) PCA and (**b**) OPLS-DA score plots of female BC (violet) and control female (orange) urine samples in the training set. (**c**) PCA and (**d**) OPLS-DA score plots of male BC (blue) and control male (green) urine samples. (**e**–**h**). The box-and-whisker plots of selected metabolites were observed in control, male, and female BC urine samples.

In both the training and validation sets, PCA and OPLS-DA score plots revealed good discrimination between separate control and cancer groups of different sex (Fig. 4a–d).

The quality factors for those models amounted to Q^2^ > 0.907 and R^2^Y > 0.986, and the *P*-values derived from the permutation tests indicated perfect discrimination of metabolite profiles between those groups. However, we did not find a significant difference between the two groups when comparing the male and female patients using the PCA scores plot (data not shown).

The performance of two models in differentiating between male and female BC patients and male and female NCs was then evaluated using ROC curve analysis. Based on the cut-off criteria (FC > 2 < 0.5, VIP > 1; AUC > 0.7, *P*-value and FDR < 0.05), finally, 79 and 48 chemical compounds appeared to be most relevant for sample distinction between male BC vs. male NCs, and female BC versus female NCs, respectively (Table S7-S8, supplementary information 2). Selected metabolites that differentiate BC patients with different gender from NCs are shown in Fig. 4e–h.

### Age-related differentiation of metabolomic profiles

**Figure 5:**
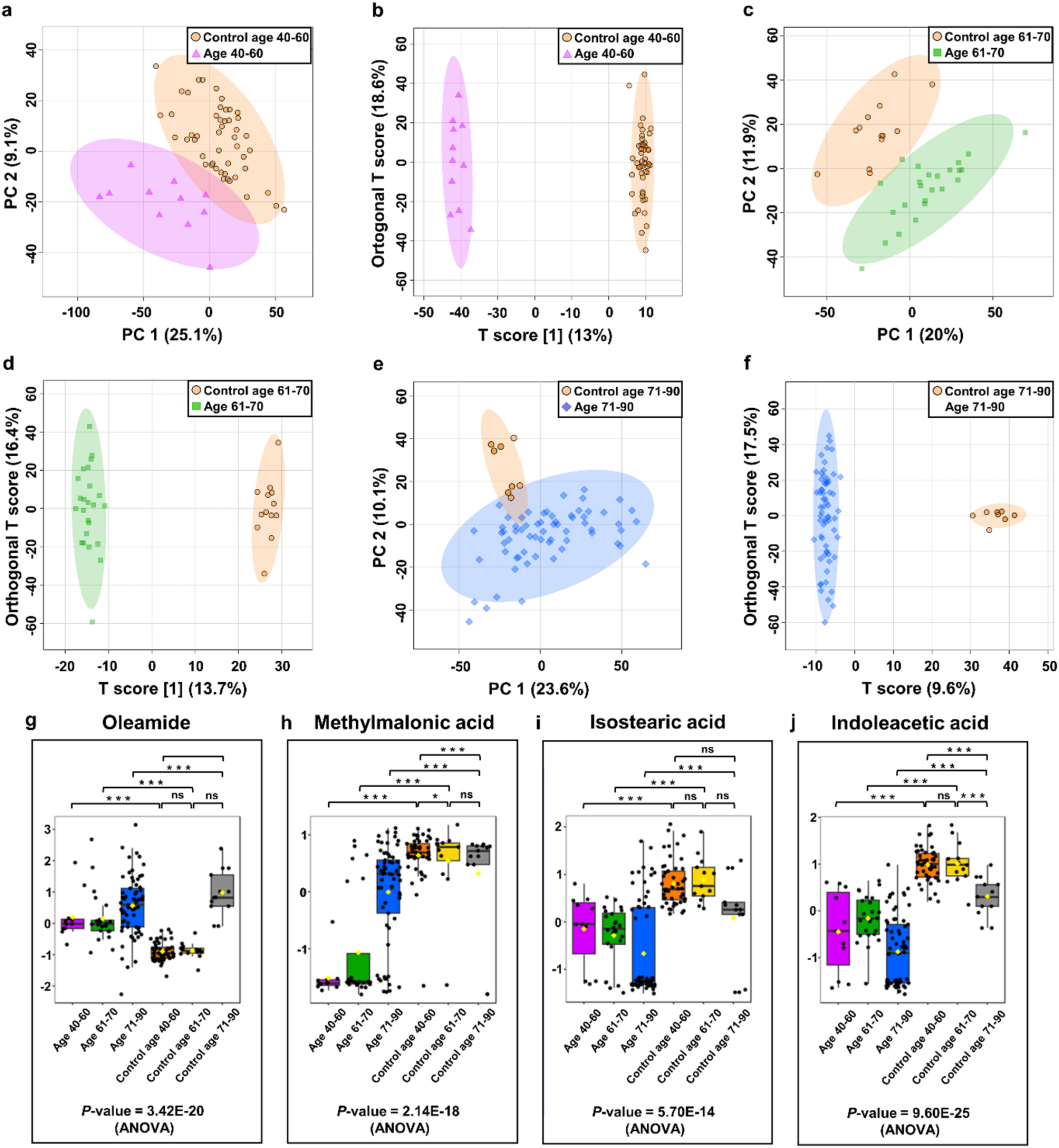
Metabolomic analysis of female/male BC and NCs of urine samples. (**a**) PCA and (**b**) OPLS-DA score plots of BC patients aged 40 to 60 (violet) and the control group aged 40 to 60 (orange) of urine samples. (**c**) PCA and (**d**) OPLS-DA score plots of BC patients aged 61 to 70 (green) and the control group aged 61 to 70 (orange) of urine samples. (**e**) PCA and (**b**) OPLS-DA score plots of BC patients aged 71 to 90 (blue) and the control group aged 71 to 90 (orange) of urine samples. (**g**–**j**) The box-and-whisker plots of selected metabolites were observed in control BC urine samples from people of different ages.

The difference in metabolites in urine extracts from patients of various ages was examined. The control group was made up of people of the same age. The entire set of LC–MS data from urine samples from BC and NCs patients was divided into three age groups. The first group consisted of 11 samples from BC patients aged 40 to 60 and 49 controls of the same age. The second group included 24 samples from BC patients aged 40 to 60 and 13 controls of the same age. The third group consisted of 65 samples from BC patients aged 40 to 60 and 13 controls of the same age. PCA and OPLS-DA score plots indicated strong age-specific identification of distinct NCs and BC groups (Fig. 5a–f).

The quality factors for those models amounted to Q^2^ > 0.867 and R^2^Y > 0.990, and the P-values derived from the permutation tests indicated perfect discrimination of metabolite profiles between those groups. The performance of three models in differentiating between BC patients of different ages and NCs was then evaluated using ROC curve analysis. Based on the cut-off criteria (FC > 2 < 0.5, VIP > 1; AUC > 0.7, *P*-value and FDR < 0.05), finally, 65, 55, and 66 chemical compounds appeared to be most relevant for sample distinction between BC patients aged 40 to 60 vs. NCs aged 40 to 60, BC patients aged 61 to 70 versus NCs aged 61 to 70 and BC patients aged 71 to 90 vs. NCs aged 71 to 90 (Table S9–S11, supplementary information 2). Selected metabolites that differentiate BC patients with different age from NCs are shown in Fig. 5g–j.

### Pathway analysis of potential biomarkers

**Figure 6:**
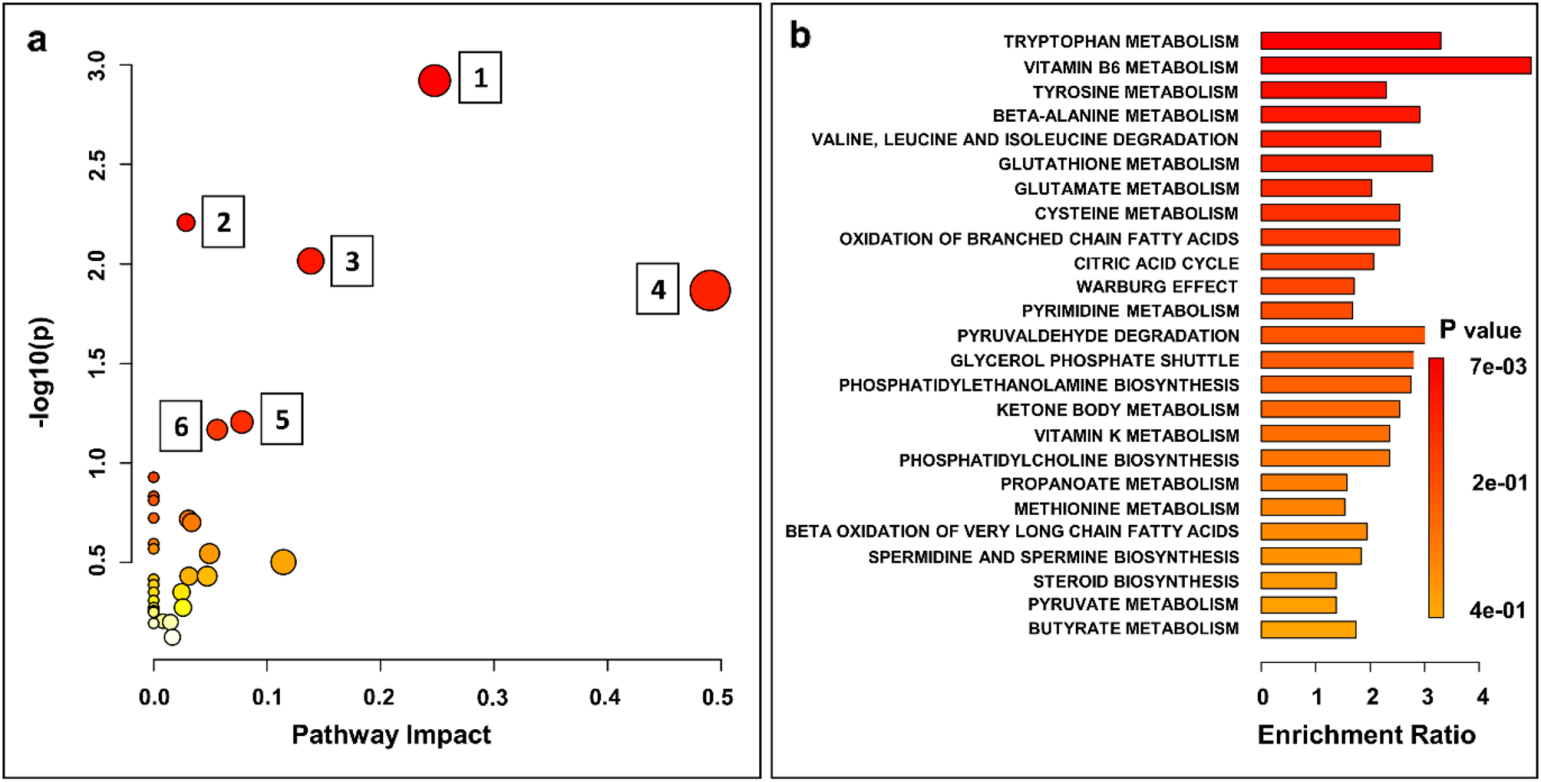
Analysis of the topology of selected statistically significant metabolites in BC. (**a**) Pathway analysis based on KEGG, with bubble area corresponding to the impact of each pathway and color representing significance from red to white, from greatest to least. (1) tryptophan metabolism; (2) pantothenate and CoA biosynthesis; (3) tyrosine metabolism (4) vitamin B6 metabolism (5) citrate cycle (TCA cycle); (6) beta-alanine metabolism; (**b**) Quantitative enrichment analysis based on SMPDB.

MetaboAnalyst 5.0 was used to perform a metabolic pathway impact analysis to identify the most relevant pathways involved in the observed changes in urine metabolite levels. Pathway and quantitative pathway enrichment analyses were performed on 116 metabolites identified in the UHPLC-UHRMS analysis. A total of 100 compounds were discovered to be relevant to human metabolism. When comparing BC to NCs, four metabolic pathways were significantly impacted (*P*-value): tryptophan metabolism, pantothenate and CoA biosynthesis, tyrosine metabolism and vitamin B6 metabolism. Figure 6a and Table S2 show the results of the pathway impact analysis (supplementary information 1).

We conducted a quantitative enrichment analysis with the MetaboAnalyst 5.0 pathway enrichment module and its associated Small Molecule Pathway Database (SMPDB) to expand the metabolomic study of bladder cancer-related pathways. Figure 6b and Table S3 show two significant pathways associated with bladder cancer: tryptophan metabolism, and vitamin B6 metabolism.

## Discussion

Over the last ten years, metabolomics studies have revealed potentially valuable information regarding the metabolic profiles of individuals afflicted with various diseases, including cancer, and possible disease progression or recurrence markers. Rapidly proliferating cancer cells have the potential to change their metabolism to suit their increased energy demands. Monitoring variations in the concentrations of various metabolites in cancer cells or bodily fluids could be a source of novel cancer biomarkers. Several studies have demonstrated the significant potential of metabolomic markers in diagnosing multiple cancers and the comprehension of the mechanisms behind cancer onset and progression^39^.

This investigation compare changes in urine metabolite levels between 100 patients with BC and 100 NCs. The 51 metabolites that distinguished these two groups the most were identified. A large group of compounds differentiating the NCs group from the BC patients (table S1, supplementary information 1) was lipids and its derivatives. Lipids serve as long-term energy storage and are the fundamental building blocks of all cell membranes. Furthermore, lipids play essential roles in living organisms, including nerve impulse transmission, hormone production and regulation, cushioning vital organs, intracellular signal transmission, and cell transporting systems. Lipid metabolism is involved in several processes related to cancer cells. Numerous studies over the last decade have shown that lipids and metabolites associated with lipid metabolism may be potential markers in human cancers, including bladder cancer^40^. We found that the urine content of 10 lipids, including four medium-chain fatty acids (2-hydroxyaproic acid, sebacic acid, azelaic acid, cis,cis-muconic acid), three acylcarnitines (3-methylglutarylcarnitine, isovalerylcarnitine, L-acetylcarnitine), two long-chain fatty acids (isostearic acid, palmitic acid) and one hydroxy fatty acid (3-hydroxymethylglutaric acid) was significantly higher in the urine of NCs than in the BC subjects. The opposite trend was observed for oleamide (Fig. 1d), which was found in much higher concentrations in the urine of BC patients compared to the NCs group, and which turned out to be the most distinguishing compound between these two groups. Of all the lipids recognized as the most differentiating, oleamide, isostearic acid, and azelaic acid with AUC > 0.9 were the most important.

Oleamide is a member of the fatty amides class of organic compounds. It is an endogenous chemical compound found naturally in blood and urine. This compound has been demonstrated to have a wide variety of neuropharmacological effects on many neurochemical systems, and it is recognized as a fatty acid amide that induces sleep^41^. Oleamide is an agonist of cannabinoid 1 and 2 (CB1 and CB2) receptors that promote cell growth and migration via adhesion and/or ionic signals at Gap junctions. Recent studies have shown that oleamide induces cell death in glioblastoma RG2 cells^42^ and inhibits Caco-2 colon cancer cell proliferation^43^. Moreover, it was also demonstrated that oleamide increases calcium ions in T24 bladder cancer cell lines, suggesting that this compound may alter the cellular function in the urinary system^44^. Oleamide has not yet been found as a possible biomarker for BC. Yet, an earlier study has revealed that the content of oleamide in the urine of patients with kidney and laryngeal cancer is greater than that of healthy people serving as controls^45,46^ and in the serum of patients with colorectal cancer^47^.

Among the lipids most differentiating cancer and the control group, there were also isostearic (Fig. 1f) and azelaic acids (Fig. 1h). Azelaic acid is a saturated nine-carbon dicarboxylic acid generated from fatty acid oxidation that suppresses neutrophil reactive oxygen species formation. Azelaic acid has been identified as a potential biomarker for colorectal cancer, with significantly lower levels in the urine of patients whit this tumor compared to healthy controls^48^. Similarly, in our studies, the urine level of azelaic acid was more deficient in BC patients than NCs (Fig. 1h).

Indoleacetic acid (IAA) was the second compound, after oleamide, to differentiate the BC group from the NCs group (Fig. 1e). IAA is a breakdown product of tryptophan metabolism in mammalian tissues that may be produced by the decarboxylation of tryptamine or the oxidative deamination of tryptophan. Some studies indicated an elevated level of IAA in the urine of patients with cervical cancer^49^ compared to controls, which may be associated with increased secretion of this compound by tumor tissues. IAA was also detected at a high level in serum samples of BC patients compared to healthy controls^50^. Our research shows a significantly lower amount of IAA in BC patients’ urine than NCs. Similar results were obtained in analyzing urinary metabolites in patients with breast cancer^51^. Moreover, indoleacetic acid is a metabolite of gut bacteria. It is possible that the changes in this metabolite were due to changes in the gut microbiome in BC patients, as previously suggested by Tan et al.^50^.

*N*-Alpha-acetyllysine is involved in DNA transcriptional activities, including the acetylation of lysine catalyzed by histone acetyltransferase enzymes by adding acetyl groups from acetyl-CoA onto lysine residues histones and nonhistone proteins. In this investigation, the level of *N*-alpha-acetyllysine in urine was higher in the BC group than in NCs individuals (Fig. 1g). This aligns with previous studies conducted by Yumba Mpanga and coworkers, 58, who identified and quantified this compound in the urine of patients with BC. Interestingly, acetyllysine was among the most statistically significant metabolites discriminating against patients with prostate cancer (PC) and healthy individuals at high-significantly lower concentrations in urine from PCa patients^52^.

To implement the proper treatment regimens for BC patients, it is required to clearly and adequately define the stage and grade of this malignancy and indicate the neoplasm. In total, 51 differential metabolites were identified as a potential markers for discriminating between LG BC patients and NCs. Indoleacetic acid (specificity—96%, sensitivity—93%) and isostearic acid (specificity—97%, sensitivity—87%) were found to be the most differentiating compounds in this model, with AUC > 0.9. Fifty-nine differential metabolites were identified as a potential marker for discriminating between HG BC patients and NCs. Among these metabolites, 5 had tremendous discriminant significance with an AUC greater than 0.95, including isostearic acid, oleamide, indoleacetic acid, 2-furoylglycine, and azelaic acid. Apart from oleamide, other compounds were identified in significantly lower levels in the urine of HG BC patients compared to NCs. 2-Furoylglycine belongs to the *N*-acyl-alpha amino acids class and is a product of fatty acid catabolism linked to mitochondrial fatty acid beta-oxidation. Earlier urine analysis of prostate cancer patients also showed significantly decreased levels of this compound in the cancer group compared to controls^53^.

Our study shows that a urine-based metabolite profile could accurately discriminate different stages of BC (pTa, pT1, and pT2) and NCs (Table 2). In the urine of patients with pTa, pT1, and pT2 stages of BC, we identified 22 the most differentiating compounds (with AUC > 0.91). One of the compounds that determined stages of BC from NCs to the greatest extent was benzaldehyde, which was identified in a much higher amount in the urine of patients from the control group. Benzaldehyde is a simple alkane whose levels rise during inflammation and oxidative stress, both of which are hallmarks of cancer^54^. In previous studies benzaldehyde was found significantly increased in BC cells lines^55^. Aldehydes are established indicators of oxidative stress and tissue damage, however in our study this does not explain the substantially lower quantities found in BC patients' urine.

**Table 2:** Differential metabolites for discrimination between pTa, pT1 and pT2 BC patients and NCs (*P*-value < 0.05; FDR < 0.05; VIP > 1; FC < 0.5 and > 2; AUC > 0.91).

No	Metabolites	Formula	*m/z*^a^	RT [min]	pTa versus control			pT1 versus control			pT2 versus control		
					FC^b^	Spec. [%]^c^	Sens. [%]^c^	FC^b^	Spec. [%]^c^	Sens. [%]^c^	FC^b^	Spec. [%]^c^	Sens. [%]^c^
1	2,5-Furandicarboxylic acid^d,e,g^	C_6_H_4_O_5_	157.0130	1.28	–	–	–	0.19	83	100	–	–	–
2	2-Furoylglycine^d,e,f,g^	C_7_H_7_NO_4_	170.0447	1.60	–	–	–	0.07	97	87	0.13	93	92
3	3,4-Dihydroxymandelic acid^d^	C_8_H_8_O_5_	226.0709	1.85	–	–	–	-	–	–	0.20	90	83
4	Azelaic acid^d,e,f,g^	C_9_H_16_O_4_	171.1014	2.54	–	–	–	0.26	79	93	0.30	93	100
5	Benzaldehyde^d^	C_7_H_6_O	107.0490	3.30	0.29	100	94	0.32	100	87	0.27	87	92
6	Choline^d^	C_5_H_14_NO	143.0702	1.81	–	–	–	0.19	81	87	–	–	–
7	*Cis*,*cis*-Muconic acid^d,e,g^	C_6_H_6_O_4_	125.0232	1.53	–	–	–	0.09	91	93	0.08	83	92
8	Indoleacetic acid^d,e,f^	C_10_H_9_NO_2_	217.0974	2.02	0.18	87	87	0.09	96	93	0.08	90	100
9	Isostearic acid^d,f,g^	C_18_H_36_O_2_	285.2785	0.20	0.14	87	87	0.20	87	87	0.47	93	92
10	Methylmalonic acid^d^	C_4_H_6_O_4_	119.0344	0.03	–	–	–	0.19	94	87	–	–	–
11	Mevalonic acid^d^	C_6_H_12_O_4_	190.1069	2.03	–	–	–	-	–	–	0.28	90	83
12	*N*-Acetylserotonin^d,e^	C_12_H_14_N_2_O_2_	175.1227	1.75	–	–	–	0.17	90	93	0.18	97	83
13	*N*-Alpha-acetyllysine^d,e,f^	C_8_H_16_N_2_O_3_	268.1056	0.14	–	–	–	14.06	87	87	–	–	–
14	Oleamide^d,f,g^	C_18_H_35_NO	282.2790	5.08	–	–	–	6.99	96	100	2.14	83	83
15	Palmitamide^d,f,g^	C_16_H_33_NO	256.2633	5.02	–	–	–	4.04	93	93	–	–	–
16	Pantothenic acid^d,f,g^	C_9_H_17_NO_5_	220.1179	1.68	–	–	–	–	–	–	0.44	90	92
17	Phenylacetylglycine^d,e,g^	C_10_H_11_NO_3_	194.0811	2.19	–	–	–	–	–	–	0.24	83	83
18	Phenylglyoxylic acid^d,e,f^	C_8_H_6_O_3_	151.0387	1.85	–	–	–	–	–	–	0.15	90	92
19	Picolinuric acid^d,f,g^	C_8_H_8_N_2_O_3_	181.0607	1.88	–	–	–	0.40	90	80	–	–	–
20	Sebacic acid^d,e,f^	C_10_H_18_O_4_	203.1275	2.22	–	–	–	0.28	81	93	0.38	83	92
21	Succinic acid^e,f^	C_4_H_6_O_4_	119.0344	0.26	–	–	–	0.46	94	80	–	–	–
22	Vanillic acid^d,e,g^	C_8_H_8_O_4_	169.0491	1.79	–	–	–	–	–	–	0.27	87	92

^a^Experimental monoisotopic mass of ion; ^b^fold change between cancer and control serum calculated from the abundance mean values for each group – cancer-to-normal ratio; ^c^ROC curve analysis for individual biomarkers; ^d^the metabolites identified by high precursor mass accuracy; ^e^the metabolites identified by matching retention time; ^f^the metabolites identified by matching isotopic pattern; ^g^the metabolites identified by matching MS/MS fragment spectra; FC: fold change; *m/z*: mass-to-charge ratio; RT: retention time; Sens.: Sensitivity; Spec.: Specificity.

Many studies have found that clinical conditions, genetic background, race, age, sex, lifestyle, diet, and medicines strongly impact the urine metabolic profile^32,56^. Age and gender are significant determinants that influence the urine metabolome, according to inter-individual variance analyses. Knowing the specific differences in metabolites linked with age and gender can give a foundation for comparative studies as well as insight into the metabolic systems of a healthy body. In literature there are evidences to suggest that sex-related differentiation can influence metabolomic profiles. Several studies have demonstrated differences in metabolomic profiles between males and females in various physiological and pathological conditions^57^. For example, a study published by Fan et al.^58^ analyzed the urine metabolome of healthy individuals and found significant sex-related differences in metabolic profiles. The study identified several metabolites that showed sex-specific variations, suggesting inherent metabolic distinctions between males and females. Another example of report of this kind was published recently^59^. The researchers identified sex-specific metabolic signatures and found that certain metabolites were significantly different between males and females, indicating potential sex-related metabolic variations. Furthermore, sex-related differences in metabolomic profiles have been observed in various diseases and conditions, including cardiovascular diseases, cancer, diabetes, and obesity. These differences may arise from variations in hormonal levels, genetic factors, and sex-specific physiological processes. However, sex-related differentiation of metabolomic profiles is a complex phenomenon influenced by multiple factors, and further research is needed to fully understand its underlying mechanisms and implications especially in BC.

Our research further explored the detailed urinary metabolites associated with BC patients of different sex. As presented in Table S3 (supplementary information 1), we have identified 19 of the most differentiating metabolites (AUC > 0.9) that most significantly determine the urine of males and females with BC from NCs. One of the compounds that the most differentiate males from the control group is tryptophan, which was found in significantly more significant amounts in urine samples from males with BC patients compared to females with BC and NCs. Tryptophan is involved in several mechanisms, including synthesizing biogenic amines such as serotonin, melatonin, and tryptamine. It contributes to the formation of nicotinamide adenine dinucleotide (NAD^+^), an essential coenzyme for energy metabolism in animals (such as the citrate cycle). Prior research suggested that tryptophan metabolism may influence human lifespan regulation.

Tryptophan metabolism has been extensively studied in relation to bladder cancer, and its significance has been consistently reported in the literature^60^. Previous studies have observed significant increases in tryptophan levels in urine, serum, and tissue samples from bladder cancer patients compared to control groups^18,61–63^. Sex-related differences have been identified in the metabolism of tryptophan, suggesting a potential link between sex hormones and tryptophan-related pathways in the context of bladder cancer^32,61^. The disruption of tryptophan metabolism in bladder cancer patients involves various mechanisms affecting enzymes and pathways. One explanation is the increased degradation of tryptophan. Bladder cancer cells may upregulate enzymes like tryptophan 2,3-dioxygenase (TDO) or indoleamine 2,3-dioxygenase (IDO), leading to heightened tryptophan degradation. This depletion reduces the availability of tryptophan for vital cellular functions^64^. Additionally, the activation of the kynurenine pathway was implicated. In bladder cancer, this pathway can become activated, resulting in the production of immunosuppressive and tumor-promoting metabolites like kynurenine, 3-hydroxykynurenine, and kynurenic acid^65^. Alterations in enzyme expression also contribute to tryptophan metabolism disruption. Changes in the levels of enzymes involved in tryptophan metabolism, such as tryptophan hydroxylase, kynurenine aminotransferases, and kynureninase, can impact the conversion of tryptophan into downstream metabolites, leading to metabolic dysregulation^66^. Immune cells like tumor-associated macrophages or regulatory T cells can stimulate the expression of IDO or TDO, resulting in tryptophan depletion and immune evasion^67^. These various mechanisms collectively contribute to the disruption of tryptophan metabolism in bladder cancer, highlighting the complexity of its involvement in the disease.

In conclusion, we show that ultra-high-resolution mass spectrometry is an effective method for characterizing urine metabolome variations in BC. We have indicated several dozen metabolites that have the potential to distinguish urine from BC patients from the urine of healthy volunteers, considering the division into different grades and stages of BC cancer as well as gender and age. To date, there is no published research indicating the specific combinations of metabolites like these proposed by our study that could potentially serve as important markers for early detection of BC. Furthermore, it was crucial to consider factors such as the stage and grade of malignancy, as well as the influence of sex and age, which can further contribute to the complexity of identifying relevant metabolomic signatures in BC. Future investigations are needed to explore these potential associations and provide a deeper understanding of the intricate interplay between metabolites, disease characteristics, and individual factors in the context of BC. Our results have the potential to help develop simple, non-invasive specific, and sensitive diagnostic tests to detect different stages and grades of BC, as well as to monitor disease recurrence.

## Supplementary Information

Supplementary Information 1.

Supplementary Tables.

## Data Availability

The corresponding author's data supporting this study’s findings are available upon reasonable request.
