# Supplementary material for: Untargeted urinary metabolomics for bladder cancer biomarker screening with ultrahigh-resolution mass spectrometry
Source: Sci Rep. 2023 Jun 16;13:9802. doi: 10.1038/s41598-023-36874-y (PMC10275937; doi:10.1038/s41598-023-36874-y)
Supplement: Supplementary file 1 — Supplementary Information 1. [file 41598_2023_36874_MOESM1_ESM.docx]

Supplementary information

**Untargeted urinary metabolomics for bladder cancer biomarker screening with ultrahigh-resolution mass spectrometry**

Joanna Nizioł^a*^, Krzysztof Ossoliński^b^, Aneta Płaza-Altamer^c^, Artur Kołodziej^c^, Anna Ossolińska^b^, Tadeusz Ossoliński^b^, Anna Nieczaj^a^, Tomasz Ruman^a^

*^a^Rzeszów University of Technology, Faculty of Chemistry, 6 Powstańców Warszawy Ave., 35-959 Rzeszów, Poland, e-mail: jniziol@prz.edu.pl*

*^b^Department of Urology, John Paul II Hospital, Grunwaldzka 4 St., 36-100 Kolbuszowa, Poland*

*^c^Doctoral School of Engineering and Technical Sciences at the Rzeszów University of Technology, 8 Powstańców Warszawy Ave., 35-959, Rzeszów, Poland*

*Corresponding author: Joanna Nizioł, e-mail: jniziol@prz.edu.pl, tel: (+48 17) 865-1896

**Table of contents**

**S1.** LC-MS data acquisition and preprocessing**2**

**Table S1.** Clinical characteristic of bladder cancer patients **3**

**Figure S1.** OPLS-DA model validation for the distinction of bladder cancer and control samples **4**

**Table S2.** Validation of the models with a sevenfold cross-validation method and permutation tests**5**

**Figure S2.** Metabolomic analysis of urine samples from HG/LG BC and NCs in the validation se**6**

**Figure S3.** Metabolomic analysis of urine samples from pTa/pT1 BC and NCs in the validation set**7**

**Table S3.** Differential metabolites for discrimination between female and male BC patients and NCs **8**

**Table S4.** Differential metabolites for discrimination between female and male BC patients and NCs**9**

**Table S3.** Result from Pathway Analysis **11**

**Table S4.** Result from Enrichment Pathway Analysis **13**

**S1****. LC-MS data acquisition and preprocessing**

Instrumental configuration consisted of a Bruker Elute UHPLC system operated by Hystar 3.3 software and a ultra-high-resolution mass spectrometer Bruker Impact II (60000+ resolution version; Bruker Daltonik GmbH) ESI QTOF-MS equipped with Data Analysis 4.2 (Bruker Daltonik GmbH), and Metaboscape (2022b). A Waters UPLC column ACQUITY BEH (C18 silica, 1.7 μm particles, 50x2.1 mm) with compatible column guard was used for all analyses. Two mobile phases were: A = water with 0.1% formic acid, B = acetonitrile with 0.1% formic acid (v/v). Samples in autosampler were thermostated at 4°C temperature. Volume of 5 μL of extract was loaded on the column at a flow rate of 200 μLmin^-1^, using 4% B. B percentage was changed with time as follows: 0 min – 1%, 0.56 min - 1% B, 4.72 min - 99%, 5.56 min - 99%, 5.6 min – 1%, 9.45 min – 1%. Solvent flow was 450 μLmin^-1^. Column was thermostated at 40°C temperature. Internal calibration on 10 mM sodium formate (in water: isopropanol 1:1 v/v) ions was performed automatically in Metaboscape with the use of syringe pump using a high precision calibration mode. All measurements were made in technical triplicates. Measurements in positive autoMSMS mode were carried out using the following parameters: *m/z* 50–1200; capillary voltage: 4.5 kV; nebulizer: 2.7 bar; dry gas: 12 L min^-1^; drying gas temperature: 220^o^C; hexapole voltage: 50 Vpp; funnel 1: 200 Vpp; funnel 2: 200 Vpp; pre-pulse storage time: 5 μs; transfer time: 60 μs. Collision-Induced Dissociation (CID) was used with following settings: absolute threshold (per 100 sum): 200 cts; absolute threshold 88 cts; active exclusion 3 spectra; release after 0.3 min, isolation mass: for *m/z* = 100, width was 3, for 500 width was 4, for 1000 was 6 and for 1300 was 8); collision energy value was 30 eV. MS frequency was 20 Hz and for MS/MS - from 5 to 30. The untargeted annotations were performed in Metaboscape (ver. 2022b) with a criterion of mass deviation (Δ*m/z*) under 2 ppm and mSigma value under 20 as the maximum acceptable deviation of the mass of the compound and the isotopic pattern respectively. For identification and molecular formula generation, exact mass of parent ions was matched with < 3 ppm error and mSigma value < 50 in most cases. All the molecular formulas were obtained using the Smart Formula tool and the C, H, N, O, P, S, Cl, Br, I and F elements. MSMS spectra was automatically matched against MSMS libraries: Bruker HMDB 2.0 (this database contains retention times that were used as additional identification factor), MassBank of North America (MoNA) library and NIST ver. 2020 MSMS library.

The quality control (QC) sample were prepared from 100 different urine extracts and were measured every ten samples throughout the analytical run to provide a set of data from which method stability and repeatability can be assessed.

**Table S1.** A data of the clinical characteristics of people with bladder cancer

| **Characteristics** | **Training set (n = 70)** | | **Validation set (n= 30)** | |
| --- | --- | --- | --- | --- |
|  | **Bladder cancer patients** | **Normal controls** | **Bladder cancer patients** | **Normal controls** |
| **No. of subjects** | 70 | 70 | 30 | 30 |
| **Age (mean/SD)** | 73/10 | 47/15 | 71/9 | 51/15 |
| **Sex** |  |  |  |  |
| Male | 55 | 51 | 26 | 19 |
| Female | 15 | 19 | 4 | 11 |
| **Grade^a^** |  |  |  |  |
| High grade | 30 | - | 12 | - |
| Low grade | 38 | - | 17 | - |
| PUNLMP | 2 | - | 1 | - |
| **Stage** |  |  |  |  |
| pTa | 47 | - | 21 | - |
| pT1 | 15 | - | 5 | - |
| pT2 | 8 | - | 4 | - |

*^a^* Tumors were classified according to World Health Organization (WHO)/ International Society of Urological Pathology (ISUP) classification criteria. LG – Low-grade; HG – high-grade; PUNLMP - papillary urothelial neoplasm of low malignant potential; pT1 and pTa – high risk non-muscle invasive bladder cancer;

pT2 – muscle invasive bladder cancer; pT- the stage has been based on pathological or microscopic findings;

**
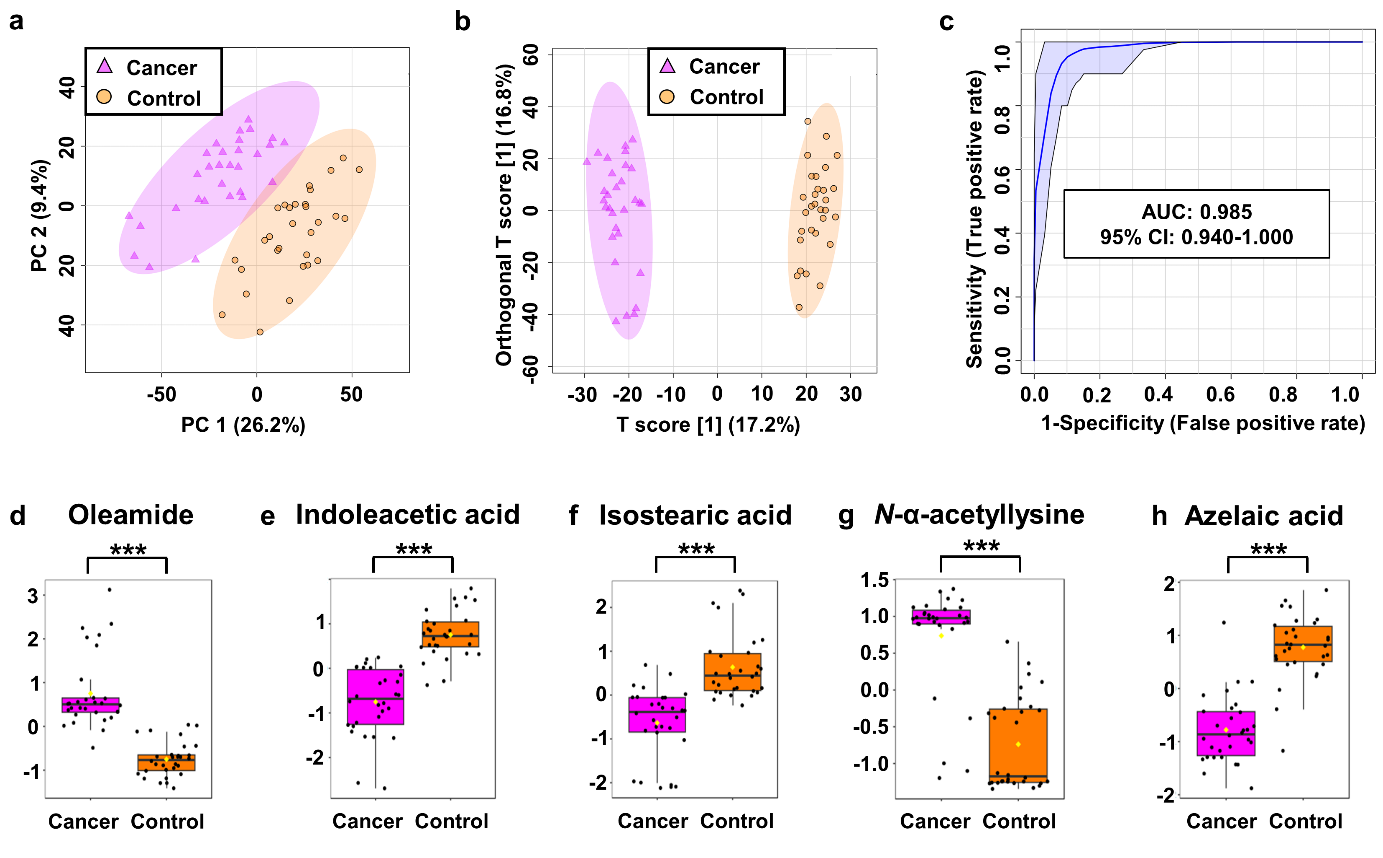
**

**Figure S1.** Metabolomic analysis of BC and NC urine samples in the validation set. (A) PCA and (B) OPLS-DA score plots of tumor (violet) and control (orange) urine samples. (C) The receiver operator characteristic (ROC) curves. (D - G) The box-and-whisker plots of selected metabolites were observed in the control and BC urine samples.

**Table S2.** Validation of the models with a sevenfold cross-validation method and permutation tests

| **Model** | **Set** | **Component** | **R^2^(X)** | **R^2^(Y)** | **Q^2^(Y)** | **Permutation**  **(2000 iternations)** | |
| --- | --- | --- | --- | --- | --- | --- | --- |
|  |  |  |  |  |  | **R^2^(Y)** | **Q^2^(Y)** |
| Cancer vs. Control | Training | P1 (Predictive) | 0.124 | 0.737 | 0.720 | 0.991 | 0.960 |
|  |  | O1 (Orthogonal in X) | 0.183 | 0.139 | 0.135 |  |  |
|  | Validation | P1 (Predictive) | 0.172 | 0.743 | 0.712 | 0.984 | 0.918 |
|  |  | O1 (Orthogonal in X) | 0.168 | 0.175 | 0.160 |  |  |
| HG vs. Control | Training | P1 (Predictive) | 0.138 | 0.778 | 0.752 | 0.994 | 0.948 |
|  |  | O1 (Orthogonal in X) | 0.179 | 0.122 | 0.118 |  |  |
|  | Validation | P1 (Predictive) | 0.153 | 0.724 | 0.666 | 0.983 | 0.879 |
|  |  | O1 (Orthogonal in X) | 0.177 | 0.187 | 0.167 |  |  |
| LG vs. Control | Training | P1 (Predictive) | 0.116 | 0.721 | 0.693 | 0.985 | 0.943 |
|  |  | O1 (Orthogonal in X) | 0.188 | 0.155 | 0.151 |  |  |
|  | Validation | P1 (Predictive) | 0.179 | 0.759 | 0.718 | 0.985 | 0.915 |
|  |  | O1 (Orthogonal in X) | 0.173 | 0.171 | 0.165 |  |  |
| pTa vs. Control | Training | P1 (Predictive) | 0.118 | 0.760 | 0.727 | 0.995 | 0.955 |
|  |  | O1 (Orthogonal in X) | 0.189 | 0.113 | 0.112 |  |  |
|  | Validation | P1 (Predictive) | 0.178 | 0.774 | 0.737 | 0.986 | 0.909 |
|  |  | O1 (Orthogonal in X) | 0.157 | 0.148 | 0.135 |  |  |
| pT1 vs. Control | Training | P1 (Predictive) | 0.129 | 0.692 | 0.620 | 0.994 | 0.914 |
|  |  | O1 (Orthogonal in X) | 0.176 | 0.204 | 0.215 |  |  |
|  | Validation | P1 (Predictive) | 0.154 | 0.664 | 0.564 | 0.985 | 0.836 |
|  |  | O1 (Orthogonal in X) | 0.198 | 0.254 | 0.222 |  |  |
| pT2 vs. Control | All set | P1 (Predictive) | 0.142 | 0.825 | 0.764 | 0.987 | 0.891 |
|  |  | O1 (Orthogonal in X) | 0.175 | 0.100 | 0.098 |  |  |
| Male vs. Control | Training | P1 (Predictive) | 0.123 | 0.728 | 0.705 | 0.990 | 0.952 |
|  |  | O1 (Orthogonal in X) | 0.185 | 0.148 | 0.145 |  |  |
|  | Validation | P1 (Predictive) | 0.161 | 0.736 | 0.692 | 0.986 | 0.907 |
|  |  | O1 (Orthogonal in X) | 0.179 | 0.187 | 0.174 |  |  |
| Female vs. Control | Training | P1 (Predictive) | 0.154 | 0.786 | 0.736 | 0.992 | 0.910 |
|  |  | O1 (Orthogonal in X) | 0.197 | 0.137 | 0.131 |  |  |
| Age 40-60 vs. Control age 40-60 | All set | P1 (Predictive) | 0.130 | 0.658 | 0.585 | 0.995 | 0.867 |
|  |  | O1 (Orthogonal in X) | 0.186 | 0.228 | 0.212 |  |  |
| Age 61-70 vs. Control age 61-70 | All set | P1 (Predictive) | 0.137 | 0.839 | 0.766 | 0.994 | 0.876 |
|  |  | O1 (Orthogonal in X) | 0.164 | 0.095 | 0.081 |  |  |
| Age 71-90 vs. Control age 71-90 | All set | P1 (Predictive) | 0.096 | 0.670 | 0.617 | 0.990 | 0.913 |
|  |  | O1 (Orthogonal in X) | 0.212 | 0.186 | 0.178 |  |  |

LG – Low-grade; HG – high-grade; pT1 and pTa – high risk non-muscle invasive bladder cancer;

pT2 – muscle invasive bladder cancer; pT- the stage has been based on pathological or microscopic findings;


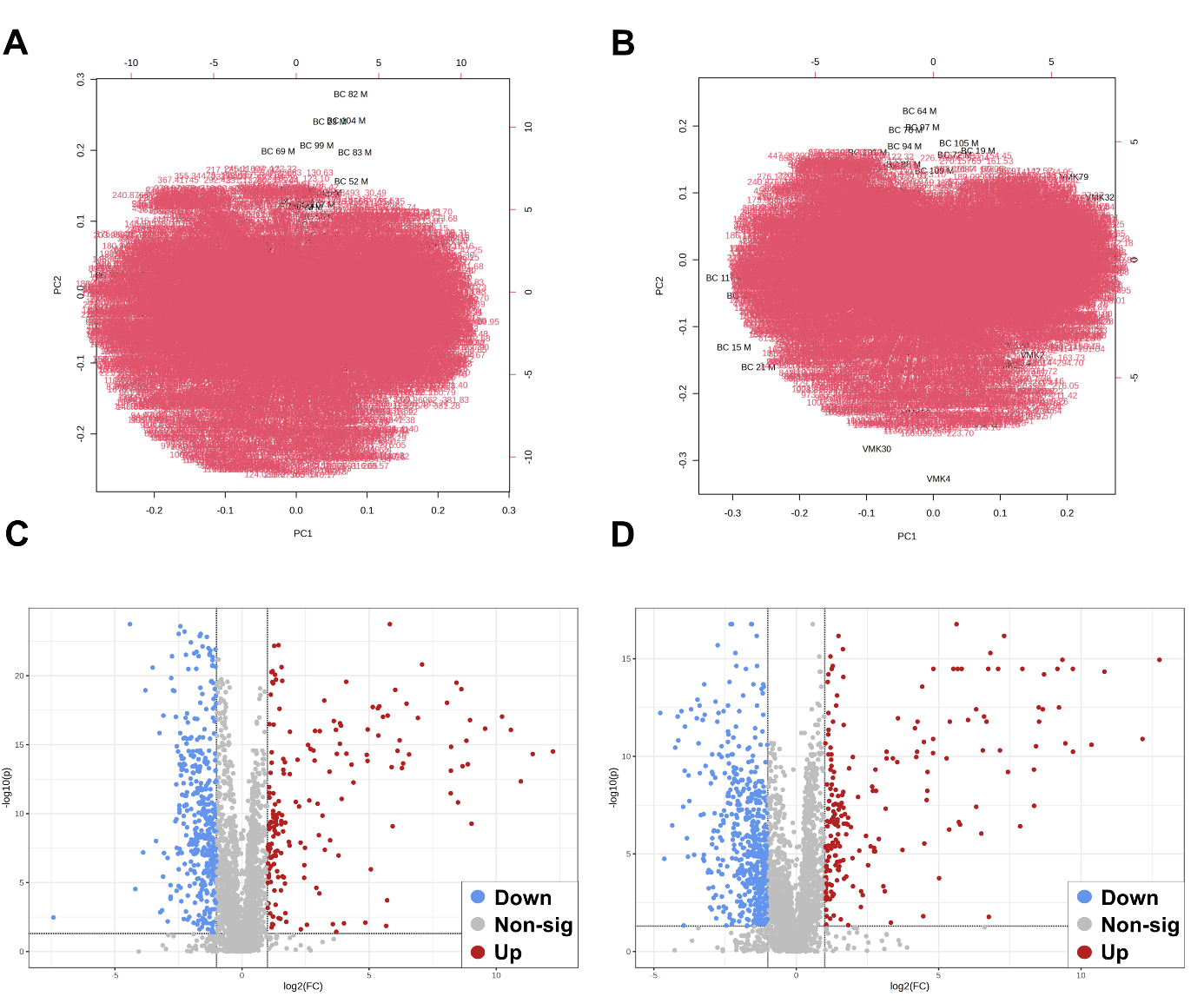


**Figure S2.** PCA biplots and volcano plots of the most significant metabolite changes comparing cancer and control group. (A, B) The PCA clustering biplots for data in (A) training and (B) validation set. (C, D) Volcano plots of the log 2-fold changes of the most significant metabolite changes comparing cancer and control group in (C) the training and (D) validation set. The red dot represented significantly upregulated metabolites, the blue dot represented significantly downregulated metabolites, and the gray dot represented not-significant metabolites

**
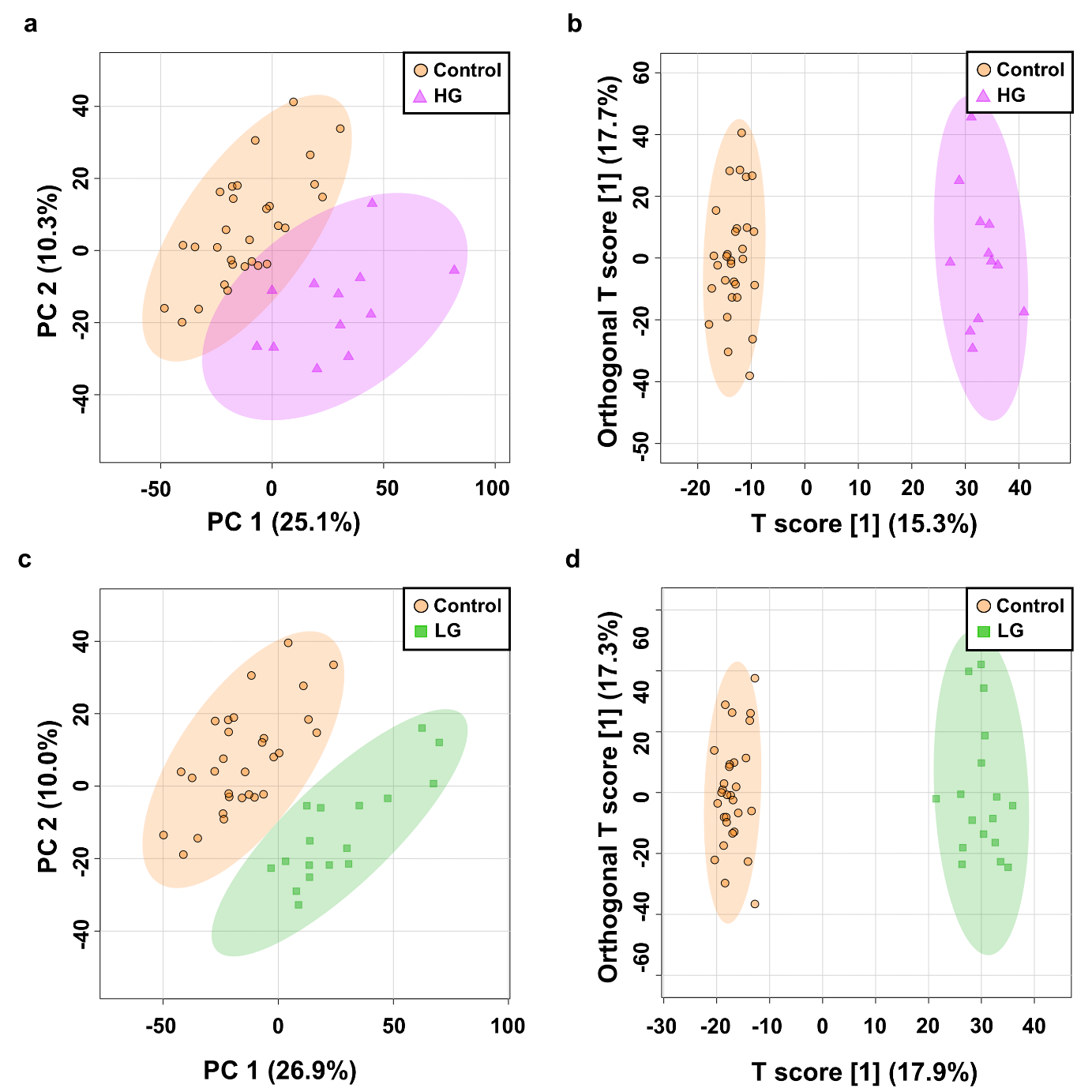
**

**Figure S3.** Metabolomic analysis of urine samples from HG/LG BC and NCs in the validation set. (A) PCA and (B) OPLS-DA score plots of HG BC (violet) and control (orange) urine samples. (C) PCA and (D) OPLS-DA score plots of LG BC (green) and control (orange) urine samples.

**
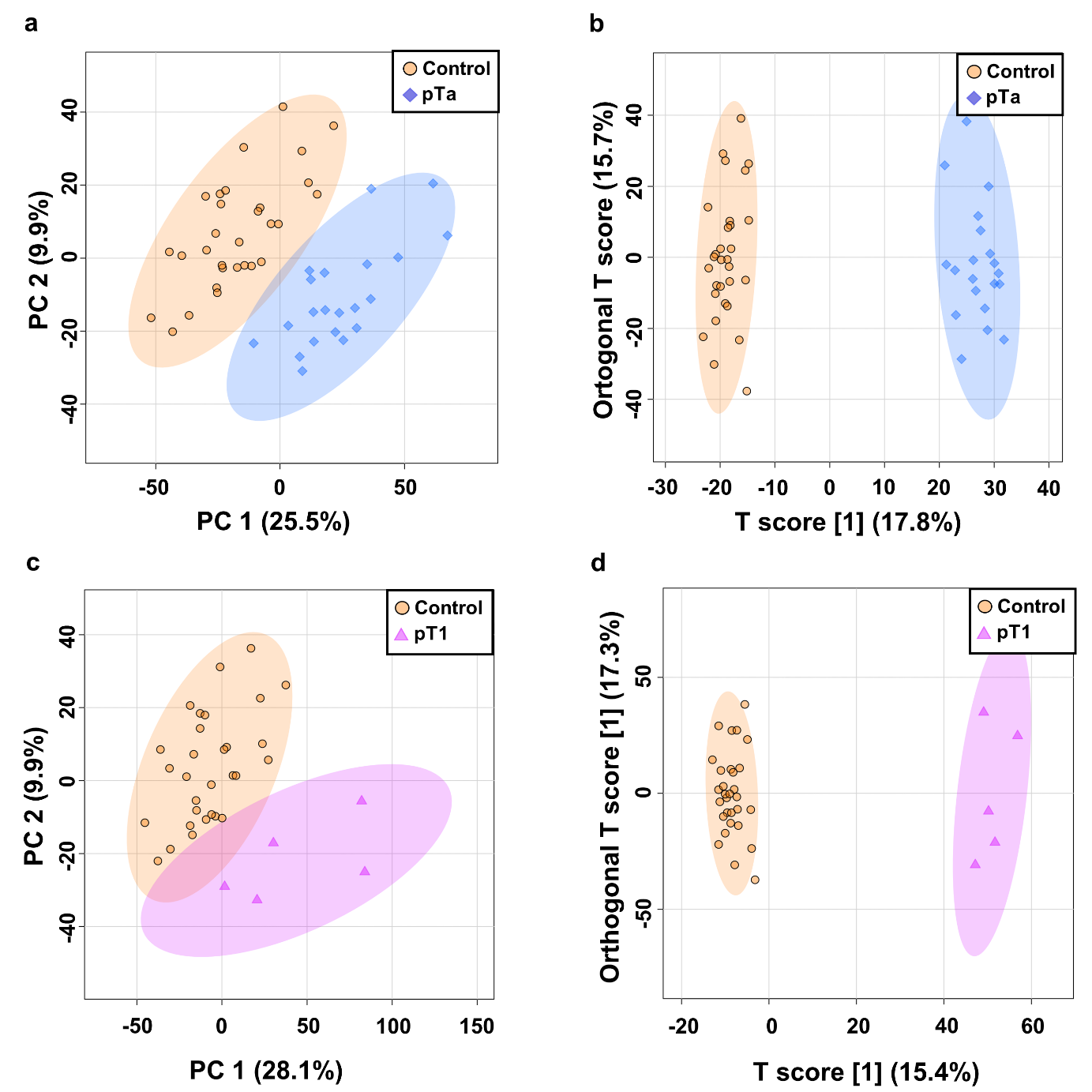
**

**Figure S4.** Metabolomic analysis of urine samples from pTa/pT1 BC and NCs in the validation set. (A) PCA and (B) OPLS-DA score plots of pTa BC (blue) and control (orange) urine samples. (C) PCA and (D) OPLS-DA score plots of pT1 BC (violet) and control (orange) urine samples.

**Table S3.** Differential metabolites for discriminating between female and male BC patients and NCs (P-value < 0.05; FDR < 0.05; VIP > 1; FC < 0.5 and > 2)

| **No.** | **Metabolites** | **Formula** | ***m/z*^a^** | **RT**  **[min]** | **Female**  **vs.**  **Control** | | | | **Male**  **vs.**  **Control** | | |
| --- | --- | --- | --- | --- | --- | --- | --- | --- | --- | --- | --- |
|  |  |  |  |  | **FC^b^** | **Spec. [%]^c^** | **Sens. [%]^c^** | **FC^b^** | | **Spec. [%]^c^** | **Sens. [%]^c^** |
| 1 | 2,5-Furandicarboxylic acid^,e,g^ | C_6_H_4_O_5_ | 157.0130 | 1.28 | 0.19 | 93 | 90 | - | | - | - |
| 2 | Azelaic acid^d,e,f,g^ | C_9_H_16_O_4_ | 171.1014 | 2.54 | 0.28 | 93 | 95 | - | | - | - |
| 3 | *Cis*,*cis*-Muconic acid^d,e,g^ | C_6_H_6_O_4_ | 125.0232 | 1.53 | 0.09 | 90 | 90 | - | | - | - |
| 4 | Deoxycytidine^d,e,f^ | C_9_H_13_N_3_O_4_ | 269.1244 | 0.69 | 0.45 | 87 | 90 | - | | - | - |
| 5 | Homovanillic acid^d,e^ | C_9_H_10_O_4_ | 183.0653 | 1.86 | 0.32 | 80 | 95 | - | | - | - |
| 6 | Indoleacetic acid^d,e,f^ | C_10_H_9_NO_2_ | 217.0974 | 2.02 | 0.08 | 90 | 100 | 0.17 | | 90 | 89 |
| 7 | Isostearic acid^d,f,g^ | C_18_H_36_O_2_ | 285.2785 | 0.20 | 0.13 | 87 | 90 | 0.14 | | 92 | 84 |
| 8 | Isovaleric acid^d,e^ | C_5_H_10_O_2_ | 144.1017 | 2.03 | 0.43 | 97 | 90 | - | | - | - |
| 9 | L-Tryptophan^d,e,f^ | C_11_H_12_N_2_O_2_ | 246.1234 | 2.04 | - | - | - | 98.62 | | 86 | 91 |
| 10 | Mevalonic acid^d^ | C_6_H_12_O_4_ | 190.1069 | 2.03 | 0.32 | 87 | 90 | - | | - | - |
| 11 | *N*-Acetylserotonin^d,e^ | C_12_H_14_N_2_O_2_ | 175.1227 | 1.75 | 0.17 | 97 | 84 | - | | - | - |
| 12 | *N*-Alpha-acetyllysine^d,e,f^ | C_8_H_16_N_2_O_3_ | 268.1056 | 0.14 | 12.50 | 90 | 84 | 16.47 | | 90 | 80 |
| 13 | Oleamide^d,f,g^ | C_18_H_35_NO | 282.2790 | 5.08 | 2.23 | 83 | 95 | 3.26 | | 90 | 91 |
| 14 | Phenylacetylglycine^d,e,g^ | C_10_H_11_NO_3_ | 194.0811 | 2.19 | 0.25 | 83 | 84 | - | | - | - |
| 15 | Phenylglyoxylic acid^d,e,f^ | C_8_H_6_O_3_ | 151.0387 | 1.85 | 0.19 | 93 | 84 | - | | - | - |
| 16 | Picolinuric acid^d,f,g^ | C_8_H_8_N_2_O_3_ | 181.0607 | 1.88 | 0.40 | 83 | 90 | - | | - | - |
| 17 | Salicyluric acid^d,e,f,g^ | C_9_H_9_NO_4_ | 196.0603 | 2.30 | 0.04 | 93 | 79 | - | | - | - |
| 18 | Succinic acid^d,e^ | C_4_H_6_O_4_ | 119.0344 | 0.26 | 0.45 | 83 | 84 | - | | - | - |
| 19 | Xanthurenic acid^d,e,f,g^ | C_10_H_7_NO_4_ | 206.0449 | 1.80 | 0.38 | 83 | 100 | - | | - | - |

^a^Experimental monoisotopic mass of ion; ^b^fold change between cancer and control urine calculated from the abundance mean values for each group – cancer-to-normal ratio; ^c^ROC curve analysis for individual biomarkers; ^d^the metabolite identified by high precursor mass accuracy; ^e^the metabolite identified by matching retention time; ^f^the metabolite identified by matching isotopic pattern; ^g^the metabolite identified by matching MS/MS fragment spectra; FC: fold change; *m/z*: mass-to-charge ratio; RT: retention time; Sens.: Sensitivity; Spec.: Specificity;

**Table S4.** Differential metabolites for discrimination between female and male BC patients and NCs (P-value < 0.05; FDR < 0.05; VIP > 1; FC < 0.5 and > 2)

| **No.** | **Metabolites** | **Formula** | ***m/z*^a^** | **RT**  **[min]** | **Age 40-60**  **vs.**  **Age control 40-60** | | | | **Age 61-70**  **vs.**  **Age control 61-70** | | | **Age 71-90**  **vs.**  **Age control 71-90** | | | |
| --- | --- | --- | --- | --- | --- | --- | --- | --- | --- | --- | --- | --- | --- | --- | --- |
|  |  |  |  |  | **FC^b^** | **Spec. [%]^c^** | **Sens. [%]^c^** | **FC^b^** | | **Spec. [%]^c^** | **Sens. [%]^c^** | **FC^b^** | **Spec. [%]^c^** | **Sens. [%]^c^** |  |
| 1 | 2,5-Furandicarboxylic acid^d,e,g^ | C_6_H_4_O_5_ | 157.0130 | 1.28 | - | - | - | - | | - | - | 0.20 | 94 | 100 |  |
| 2 | 2-Furoic acid^d,e,f^ | C_5_H_4_O_3_ | 130.0498 | 1.42 | - | - | - | 0.11 | | 79 | 100 | - | - | - |  |
| 3 | 2-Furoylglycine^d,e,f,g^ | C_7_H_7_NO_4_ | 170.0447 | 1.60 | 0.15 | 91 | 78 | 0.22 | | 88 | 92 | 0.35 | 88 | 92 |  |
| 4 | 2-Hydroxycaproic acid^d,e,f^ | C_6_H_12_O_3_ | 174.1122 | 2.10 | - | - | - | - | | - | - | 0.20 | 83 | 85 |  |
| 5 | 3,4-Dihydroxymandelic acid^d^ | C_8_H_8_O_5_ | 226.0709 | 1.85 | - | - | - | 0.18 | | 92 | 100 | - | - | - |  |
| 6 | 3-Hydroxymethylglutaric acid^d,e^ | C_6_H_10_O_5_ | 185.0418 | 0.43 | - | - | - | - | | - | - | 0.16 | 85 | 77 |  |
| 7 | 4-Ethylphenol^d,e,f^ | C_8_H_10_O | 164.1069 | 2.48 | - | - | - | 49.40 | | 96 | 85 | - | - | - |  |
| 8 | Acetylcysteine^d,e^ | C_5_H_9_NO_3_S | 164.0374 | 1.85 | - | - | - | - | | - | - | 0.23 | 86 | 92 |  |
| 9 | Alpha-ketoisovaleric acid^d,e^ | C_5_H_8_O_3_ | 158.0811 | 1.65 | 0.40 | 91 | 78 | - | | - | - | - | - | - |  |
| 10 | Anserine^d,e,g^ | C_10_H_16_N_4_O_3_ | 241.1295 | 0.35 | - | - | - | 0.21 | | 88 | 100 | - | - | - |  |
| 11 | Azelaic acid^d,e,f,g^ | C_9_H_16_O_4_ | 171.1014 | 2.54 | 0.28 | 91 | 90 | - | | - | - | 0.47 | 92 | 92 |  |
| 12 | Cis,cis-Muconic acid^d,e,g^ | C_6_H_6_O_4_ | 125.0232 | 1.53 | - | - | - | 0.12 | | 96 | 85 | 0.13 | 83 | 100 |  |
| 13 | Gamma-Glutamylcysteine^d,f^ | C_8_H_14_N_2_O_5_S | 251.0701 | 4.18 | - | - | - | 4.37 | | 83 | 92 | - | - | - |  |
| 14 | Indoleacetic acid^d,e,f^ | C_10_H_9_NO_2_ | 217.0974 | 2.02 | - | - | - | 0.08 | | 96 | 92 | 0.20 | 92 | 100 |  |
| 15 | Isobutyryl-L-carnitine^d,f,g^ | C_11_H_21_NO_4_ | 232.1543 | 1.72 | 0.35 | 91 | 82 | - | | - | - | 0.33 | 86 | 92 |  |
| 16 | Isostearic acid^d,f,g^ | C_18_H_36_O_2_ | 285.2788 | 0.20 | - | - | - | 0.09 | | 92 | 92 | 0.10 | 82 | 100 |  |
| 17 | Leu-Gln^d,f,g^ | C_11_H_21_N_3_O_4_ | 260.1604 | 1.58 | - | - | - | 18.58 | | 100 | 77 | - | - | - |  |
| 18 | L-Thyronine^d,e,f^ | C_15_H_15_NO_4_ | 312.0647 | 1.94 | - | - | - | 186.02 | | 92 | 92 | - | - | - |  |
| 19 | L-Tryptophan^d,e,f^ | C_11_H_12_N_2_O_2_ | 246.1237 | 2.04 | - | - | - | - | | - | - | 4332.60 | 100 | 77 |  |
| 20 | Methylhippuric acid^d,e,f^ | C_10_H_11_NO_3_ | 235.1076 | 2.36 | 0.10 | 91 | 80 | - | | - | - | - | - | - |  |
| 21 | Methylmalonic acid^d^ | C_4_H_6_O_4_ | 119.0344 | 0.03 | 0.02 | 100 | 98 | - | | - | - | - | - | - |  |
| 22 | N-Acetyl-L-tyrosine^d,e^ | C_11_H_13_NO_4_ | 241.1180 | 1.95 | 0.26 | 82 | 78 | - | | - | - | - | - | - |  |
| 23 | N-Acetylserotonin^d,e^ | C_12_H_14_N_2_O_2_ | 175.1227 | 1.75 | 0.23 | 91 | 84 | - | | - | - | - | - | - |  |
| 24 | N-Alpha-acetyllysine^d,e,f^ | C_8_H_16_N_2_O_3_ | 268.1056 | 0.14 | 16.46 | 91 | 100 | 24.43 | | 88 | 100 | 109.77 | 100 | 92 |  |
| 25 | Oleamide^d,f,g^ | C_18_H_35_NO | 282.2790 | 5.08 | 2.48 | 91 | 96 | 2.49 | | 92 | 92 | 3.14 | 97 | 100 |  |
| 26 | Palmitamide^d,f,g^ | C_16_H_33_NO | 256.2633 | 5.02 | - | - | - | 2.16 | | 88 | 100 |  |  |  |  |
| 27 | Phenylacetylglycine^d,e,g^ | C_10_H_11_NO_3_ | 194.0811 | 2.19 | 0.22 | 82 | 90 | 0.22 | | 83 | 85 | 0.25 | 85 | 100 |  |
| 28 | Phenylglyoxylic acid^d,e,f^ | C_8_H_6_O_3_ | 151.0387 | 1.85 | 0.25 | 73 | 100 | 0.17 | | 92 | 100 | 0.37 | 94 | 77 |  |
| 29 | Picolinuric acid^d,f,g^ | C_8_H_8_N_2_O_3_ | 181.0607 | 1.88 | 0.45 | 82 | 90 | - | | - | - | 0.35 | 88 | 92 |  |
| 30 | Sebacic acid^d,e,f^ | C_10_H_18_O_4_ | 203.1275 | 2.22 | 0.25 | 91 | 90 | - | | - | - | - | - | - |  |
| 31 | Succinic acid^d,e^ | C_4_H_6_O_4_ | 119.0344 | 0.26 | 0.40 | 91 | 90 | - | | - | - | - | - | - |  |
| 32 | Vanillic acid^d,f,g^ | C_8_H_8_O_4_ | 169.0491 | 1.79 | - | - | - | 0.25 | | 88 | 92 | - | - | - |  |
| 33 | Xanthurenic acid^d,e,f,g^ | C_10_H_7_NO_4_ | 206.0449 | 1.80 | - | - | - | - | | - | - | 0.29 | 88 | 92 |  |

^a^Experimental monoisotopic mass; ^b^fold change between cancer and control urine calculated from the abundance mean values for each group – cancer-to-normal ratio; ^c^ROC curve analysis for individual biomarkers; ^d^the metabolite identified by high precursor mass accuracy; ^e^the metabolite identified by matching retention time; ^f^the metabolite identified by matching isotopic pattern; ^g^the metabolite identified by matching MS/MS fragment spectra; FC: fold change; *m/z*: mass-to-charge ratio; RT: retention time; Sens.: Sensitivity; Spec.: Specificity;

**Table S5.** Result from Pathway Analysis with P-value > 0.05

| **KEGG pathway** | **Total**^a^ | **Hits**^b^ | **Impact**^c^ | ***P-*value**^d^ | | **Holm p**^e^ | | **FDR**^f^ | |
| --- | --- | --- | --- | --- | --- | --- | --- | --- | --- |
| Tryptophan metabolism | 41 | 5 | 0.248 | 0.001 | 0.101 | | 0.101 | |  |
| Pantothenate and CoA biosynthesis | 19 | 3 | 0.029 | 0.006 | 0.514 | | 0.260 | |  |
| Tyrosine metabolism | 42 | 4 | 0.138 | 0.010 | 0.792 | | 0.270 | |  |
| Vitamin B6 metabolism | 9 | 2 | 0.490 | 0.014 | 1.000 | | 0.285 | |  |
| Citrate cycle (TCA cycle) | 20 | 2 | 0.078 | 0.062 | 1.000 | | 0.951 | |  |
| beta-Alanine metabolism | 21 | 2 | 0.056 | 0.068 | 1.000 | | 0.951 | |  |
| D-Glutamine and D-glutamate metabolism | 6 | 1 | 0.000 | 0.118 | 1.000 | | 1.000 | |  |
| Glycine, serine and threonine metabolism | 33 | 2 | 0.000 | 0.147 | 1.000 | | 1.000 | |  |
| Valine, leucine and isoleucine biosynthesis | 8 | 1 | 0.000 | 0.154 | 1.000 | | 1.000 | |  |
| Phenylalanine metabolism | 10 | 1 | 0.000 | 0.189 | 1.000 | | 1.000 | |  |
| Pyrimidine metabolism | 39 | 2 | 0.031 | 0.191 | 1.000 | | 1.000 | |  |
| Valine, leucine and isoleucine degradation | 40 | 2 | 0.033 | 0.199 | 1.000 | | 1.000 | |  |
| Arginine biosynthesis | 14 | 1 | 0.000 | 0.254 | 1.000 | | 1.000 | |  |
| Butanoate metabolism | 15 | 1 | 0.000 | 0.270 | 1.000 | | 1.000 | |  |
| Histidine metabolism | 16 | 1 | 0.049 | 0.285 | 1.000 | | 1.000 | |  |
| Terpenoid backbone biosynthesis | 18 | 1 | 0.114 | 0.314 | 1.000 | | 1.000 | |  |
| Pyruvate metabolism | 22 | 1 | 0.031 | 0.370 | 1.000 | | 1.000 | |  |
| Pentose phosphate pathway | 22 | 1 | 0.047 | 0.370 | 1.000 | | 1.000 | |  |
| Propanoate metabolism | 23 | 1 | 0.000 | 0.383 | 1.000 | | 1.000 | |  |
| Lysine degradation | 25 | 1 | 0.000 | 0.409 | 1.000 | | 1.000 | |  |
| Alanine, aspartate and glutamate metabolism | 28 | 1 | 0.000 | 0.445 | 1.000 | | 1.000 | |  |
| Glutathione metabolism | 28 | 1 | 0.025 | 0.445 | 1.000 | | 1.000 | |  |
| Glyoxylate and dicarboxylate metabolism | 32 | 1 | 0.000 | 0.491 | 1.000 | | 1.000 | |  |
| Biosynthesis of unsaturated fatty acids | 36 | 1 | 0.000 | 0.532 | 1.000 | | 1.000 | |  |
| Glycerophospholipid metabolism | 36 | 1 | 0.026 | 0.532 | 1.000 | | 1.000 | |  |
| Arginine and proline metabolism | 38 | 1 | 0.000 | 0.552 | 1.000 | | 1.000 | |  |
| Fatty acid elongation | 39 | 1 | 0.000 | 0.561 | 1.000 | | 1.000 | |  |
| Fatty acid degradation | 39 | 1 | 0.000 | 0.561 | 1.000 | | 1.000 | |  |
| Primary bile acid biosynthesis | 46 | 1 | 0.008 | 0.622 | 1.000 | | 1.000 | |  |
| Fatty acid biosynthesis | 47 | 1 | 0.015 | 0.630 | 1.000 | | 1.000 | |  |
| Aminoacyl-tRNA biosynthesis | 48 | 1 | 0.000 | 0.638 | 1.000 | | 1.000 | |  |
| Purine metabolism | 65 | 1 | 0.017 | 0.750 | 1.000 | | 1.000 | |  |

^a^The total number of compounds in the pathway; ^b^the hits is the actually matched number from the NMR and data; ^c^the pathway impact value calculated from pathway topology analysis; ^d^*P-*value calculated from the enrichment analysis; ^e^*P-*value adjusted by Holm–Bonferroni method; ^f^*P-*value adjusted using False Discovery Rate; KEGG: Kyoto Encyclopedia of Genes and Genomes

**Table S6.** Result from Enrichment Pathway Analysis with *P*-value

| **SMPDB pathway** | **Total**^a^ | **Hits**^b^ | **Expected**^c^ | ***P-*value**^d^ | **Holm p**^e^ | **FDR**^f^ |
| --- | --- | --- | --- | --- | --- | --- |
| Tryptophan Metabolism | 60 | 6 | 1.820 | 0.007 | 0.718 | 0.718 |
| Vitamin B6 Metabolism | 20 | 3 | 0.605 | 0.020 | 1.000 | 0.990 |
| Tyrosine Metabolism | 72 | 5 | 2.180 | 0.060 | 1.000 | 1.000 |
| Beta-Alanine Metabolism | 34 | 3 | 1.030 | 0.080 | 1.000 | 1.000 |
| Valine, Leucine and Isoleucine Degradation | 60 | 4 | 1.820 | 0.102 | 1.000 | 1.000 |
| Glutathione Metabolism | 21 | 2 | 0.636 | 0.130 | 1.000 | 1.000 |
| Glutamate Metabolism | 49 | 3 | 1.480 | 0.182 | 1.000 | 1.000 |
| Cysteine Metabolism | 26 | 2 | 0.787 | 0.184 | 1.000 | 1.000 |
| Oxidation of Branched Chain Fatty Acids | 26 | 2 | 0.787 | 0.184 | 1.000 | 1.000 |
| Citric Acid Cycle | 32 | 2 | 0.969 | 0.252 | 1.000 | 1.000 |
| Warburg Effect | 58 | 3 | 1.760 | 0.254 | 1.000 | 1.000 |
| Pyrimidine Metabolism | 59 | 3 | 1.790 | 0.263 | 1.000 | 1.000 |
| Pyruvaldehyde Degradation | 10 | 1 | 0.303 | 0.266 | 1.000 | 1.000 |
| Glycerol Phosphate Shuttle | 11 | 1 | 0.333 | 0.288 | 1.000 | 1.000 |
| Phosphatidylethanolamine Biosynthesis | 12 | 1 | 0.363 | 0.310 | 1.000 | 1.000 |
| Ketone Body Metabolism | 13 | 1 | 0.394 | 0.331 | 1.000 | 1.000 |
| Vitamin K Metabolism | 14 | 1 | 0.424 | 0.352 | 1.000 | 1.000 |
| Phosphatidylcholine Biosynthesis | 14 | 1 | 0.424 | 0.352 | 1.000 | 1.000 |
| Propanoate Metabolism | 42 | 2 | 1.270 | 0.366 | 1.000 | 1.000 |
| Methionine Metabolism | 43 | 2 | 1.300 | 0.378 | 1.000 | 1.000 |
| Beta Oxidation of Very Long Chain Fatty Acids | 17 | 1 | 0.515 | 0.410 | 1.000 | 1.000 |
| Spermidine and Spermine Biosynthesis | 18 | 1 | 0.545 | 0.428 | 1.000 | 1.000 |
| Steroid Biosynthesis | 48 | 2 | 1.450 | 0.432 | 1.000 | 1.000 |
| Pyruvate Metabolism | 48 | 2 | 1.450 | 0.432 | 1.000 | 1.000 |
| Butyrate Metabolism | 19 | 1 | 0.575 | 0.445 | 1.000 | 1.000 |
| Mitochondrial Electron Transport Chain | 19 | 1 | 0.575 | 0.445 | 1.000 | 1.000 |

^a^The total number of compounds in the pathway; ^b^the hits is the actually matched number from the NMR data; ^c^the pathway impact value calculated from pathway topology analysis; ^d^*P-*value calculated from the enrichment analysis; ^e^*P-*value adjusted by Holm–Bonferroni method; ^f^*P-*value adjusted using False Discovery Rate; SMPDB: The Small Molecule Pathway Database
